# Bi‐Induced Few‐Layered Graphite Frameworks as Efficient Interfacial Transitions Toward Ultrafast Potassium Storage

**DOI:** 10.1002/advs.202416742

**Published:** 2025-04-15

**Authors:** Bozhi Yang, Xin Min, Xinyu Zhu, Shaorou Ke, Shujie Yang, Ya Chen, Wei Wang, Ruiyu Mi, Yangai Liu, Zhaohui Huang, Xi Kai, Minghao Fang, R. Vasant Kumar

**Affiliations:** ^1^ Engineering Research Center of Ministry of Education for Geological Carbon Storage and Low Carbon Utilization of Resources Beijing Key Laboratory of Materials Utilization of Nonmetallic Minerals and Solid Wasters National Laboratory of Mineral Materials School of Materials Science and Technology China University of Geoscience (Beijing) Beijing 100083 P. R. China; ^2^ State Key Laboratory of Advanced Metallurgy School of Metallurgical and Ecological Engineering University of Science and Technology Beijing Beijing 100083 P. R. China; ^3^ Department of Applied Chemistry School of Chemistry University Engineering Research Center of Energy Storage Materials and Chemistry of Shaanxi Province State Key Laboratory for Electrical Insulation and Power Equipment Xi'an Jiaotong University Xi'an 710049 P. R. China; ^4^ Department of Materials Science and Metallurgy University of Cambridge Cambridge CB3 0FS UK

**Keywords:** Bi‐induced few‐layered graphite, bismuth anode, interfacial transition, potassium ion battery

## Abstract

Bismuth is a promising anode material for potassium‐ion batteries due to its green, non‐toxic and high theoretical capacity (384 mAh g^−1^). However, the sluggish reaction kinetics and excessive volume expansion during cycling limit its practical application. Herein, Bi‐induced few‐layered graphite frameworks are in situ encapsulated on the surface of Bi nanoparticles, based on the mechanism of graphitization by rearrangement of interstitial carbon atoms during the nucleation process of Bi, while these composite particles are embedded in Bi‐doped porous carbon fibers composite. The graphite frameworks can stabilize the structure while serving as an efficient interfacial transfer layer, enabling rapid transport of both potassium ions and electrons. Bi atoms doped into the carbon fiber matrix effectively enhances the potassium ion transport kinetics in amorphous carbon by lowering the migration energy barrier of potassium ions in the carbon layer. The porous structure effectively alleviates the volume expansion of Bi nanoparticles during cycling, which synergistically results in superior high‐rate performance and cycling stability. Finally, the capacity can reach 215 mAh g^−1^ at 10 A g^−1^, and a capacity retention rate of 83.8% is achieved after 6000 cycles at 10 A g^−1^ with an ultra‐low decay rate of 0.00278% per cycle.

## Graphical abstract

Design and synthesis of a composite anode material for potassium‐ion batteries, featuring few‐layered graphite encapsulated Bi nanoparticles embedded in Bi‐doped porous carbon fibers, to achieve ultrafast potassium storage. The few‐layer graphene framework, derived from a graphitization mechanism involving interstitial carbon atom rearrangement during Bi nucleation, promotes rapid potassium‐ion and electron transport.

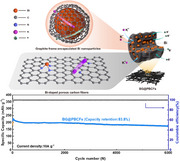

## 1 Introduction

The rapid development of renewable energy effectively alleviates energy pressure, while the intermittent of renewable energy sources stimulates the demand for large‐scale and high‐performance energy storage device.^[^ ^1^ ^]^ Since its inception, lithium‐ion batteries (LIBs) have received widespread attention in the market. Currently, rechargeable lithium‐ion batteries dominate the portable energy storage market.^[^ ^2^ ^]^ However, facing the increasing demand for large‐scale energy storage, the demand for lithium resources has become increasingly strong and further leads to the scarcity and rising cost of lithium resources. Instead, potassium‐ion batteries (PIBs), with a high crustal abundance and low cost, are currently a highly potential alternative technology.^[^ ^3^ ^]^ However, the larger ionic radius of potassium ions would lead to severe volume expansion of anode materials after K^+^ intercalation, causing material fragmentation and poor electrochemical performance.^[^ ^4^ ^]^ Therefore, the design and development of superior anode materials is a focal point of research aimed at promoting the application of potassium‐ion batteries in the field of energy storage.

The anode materials of PIBs can be classified into intercalation‐, conversion‐, and alloying‐type based on their K^+^ storage mechanisms.^[^ ^3b^ ^]^ Intercalation‐type materials mainly include graphite, hard carbon, and soft carbon.^[^ ^5^ ^]^ Graphite, when used as anode material for PIBs, shows a theoretical capacity of 279 mAh g^−1^. Although it has been experimentally proven that K^+^ can be reversibly intercalated/deintercalated into/from the graphite interlayer, its capacity, rate performance, and cycling performance for PIBs are not satisfactory. Conversion‐type materials, mainly including some metal oxides, sulfides, phosphides, etc., such as SnO_2_, Sb_2_S_3_, FeP, etc., usually have high theoretical capacity.^[^ ^6^ ^]^ However, they suffer from large volume changes during the intercalation/deintercalation of K^+^, resulting in capacity loss and poor reversibility. Alloying‐type materials are mainly composed of Group IV and V elements as metal elements or their alloys, such as Sb, Sn, Bi, SnSb, BiSb, etc.^[^ ^7^ ^]^ Due to their high theoretical capacity, researchers mostly focus on improving the alloying process with K under appropriate voltage plateaus.

Among them, the metal Bi anodes, with environmental friendliness and low cost, have been widely studied. The unique large lattice spacing (d (003) = 0.395 nm along the c‐axis) and high theoretical capacity of 384 mAh g^−1^ endow it with excellent potassium storage capability.^[^ ^7^, ^8^ ^]^ However, during the alloying/dealloying process, the significant volume change and slow kinetics hinder its large‐scale application. Researchers have used various strategies to improve these drawbacks, including interface regulation, size regulation, structural design, and material composites. Regulating electrolyte type is an effective interface regulation strategy to improve the potassium storage performance of Bi. The researchers found that ether‐based electrolytes in Bi‐based electrodes can form elastic and firm SEI layers compared to carbonate‐based electrolytes to ensure the best electrode/electrolyte interface and effectively adapt to the structural changes caused by Bi metal volume expansion.^[^ ^9^ ^]^ Appropriate size control can shorten the ion diffusion path and buffer volume expansion. Cheng et al. clarified the morphological evolution of Bi metal anodes with 0D, 1D, 2D, and 3D structures during K^+^ intercalation/deintercalation, among which 2D‐Bi achieved excellent long‐term cycling stability and outstanding rate performance.^[^ ^8c^ ^]^ Experimental results have shown that the combination of unique structural design and carbon material composite strategy can effectively inhibit volume expansion and form a conductive carbon network to obtain excellent cycling and rate performance. Liu et al. designed a unique spindle‐shaped structure of Bi@N‐doped carbon composite material (SPB@NC), which has excellent durable cycling performance due to its interconnected hollow nano Bi and hard carbon layer composite structure.^[^ ^10^ ^]^ Cui et al. designed a mixed ball cactus‐shaped bismuth nanosphere embedded in a 3D nitrogen‐rich carbon nanonetwork, which gradually transformed into a 3D porous nanonetwork during cycling, improving the utilization of active materials.^[^ ^11^ ^]^ However, integrating the above strategies into a Bi‐based PIBs anodes material and employing a facile process to achieve high capacity, excellent performance, and long cycle life remains a formidable challenge.

In this work, we have designed a few‐layered graphite encapsulated Bi nanoparticles embedded in Bi‐doped porous carbon fibers composite (BG@PBCFs). In this unique structure, Bi nanoparticles induce the graphitization of the surrounding amorphous carbon to form the few‐layered graphite frameworks, which serves as an efficient transport interface. This interface, in conjunction with the Bi‐doped carbon nanofibers, can synergistically facilitate the diffusion of potassium ions toward Bi nanoparticles. The few‐layered graphite frameworks that encapsulates Bi nanoparticles can effectively stabilize their structure via a sliding process between adjacent graphite layers, while the porous carbon fiber's channel structure offers an abundant volumetric buffer space. This dual carbon layers structural stabilization strategy can significantly mitigate the volumetric expansion of Bi during the intercalation and deintercalation of potassium ions. The large specific surface area and 3D porous fiber morphology of the BG@PBCFs structure increase the available electrode‐electrolyte contact interface, shortening the diffusion pathway of potassium ions, thereby improving its rate performance. Benefitting from the unique few‐layered graphite frameworks and Bi‐doped porous carbon nanofiber structure, the obtained BG@PBCFs electrode exhibits outstanding rate performance of 215 mAh g^−1^ at 10 A g^−1^ and ultralong cycling life (a capacity retention rate of 83.8% after more than 6000 cycles at 10 A g^−1^ and an ultra‐low decay rate of 0.00278% per cycle.) as an anode of PIBs. The full cell assembled with this anode also demonstrates outstanding rate performance, delivering a specific capacity of 144 mAh g^−1^ at 10 A g^−1^ and a maximum energy density of 221 Wh kg^−1^ at 528 W kg^−1^, indicating its potential for practical applications.

## 2 Results and Discussion

### 2.1 Material Synthesis and Characterization

**Scheme 1:**
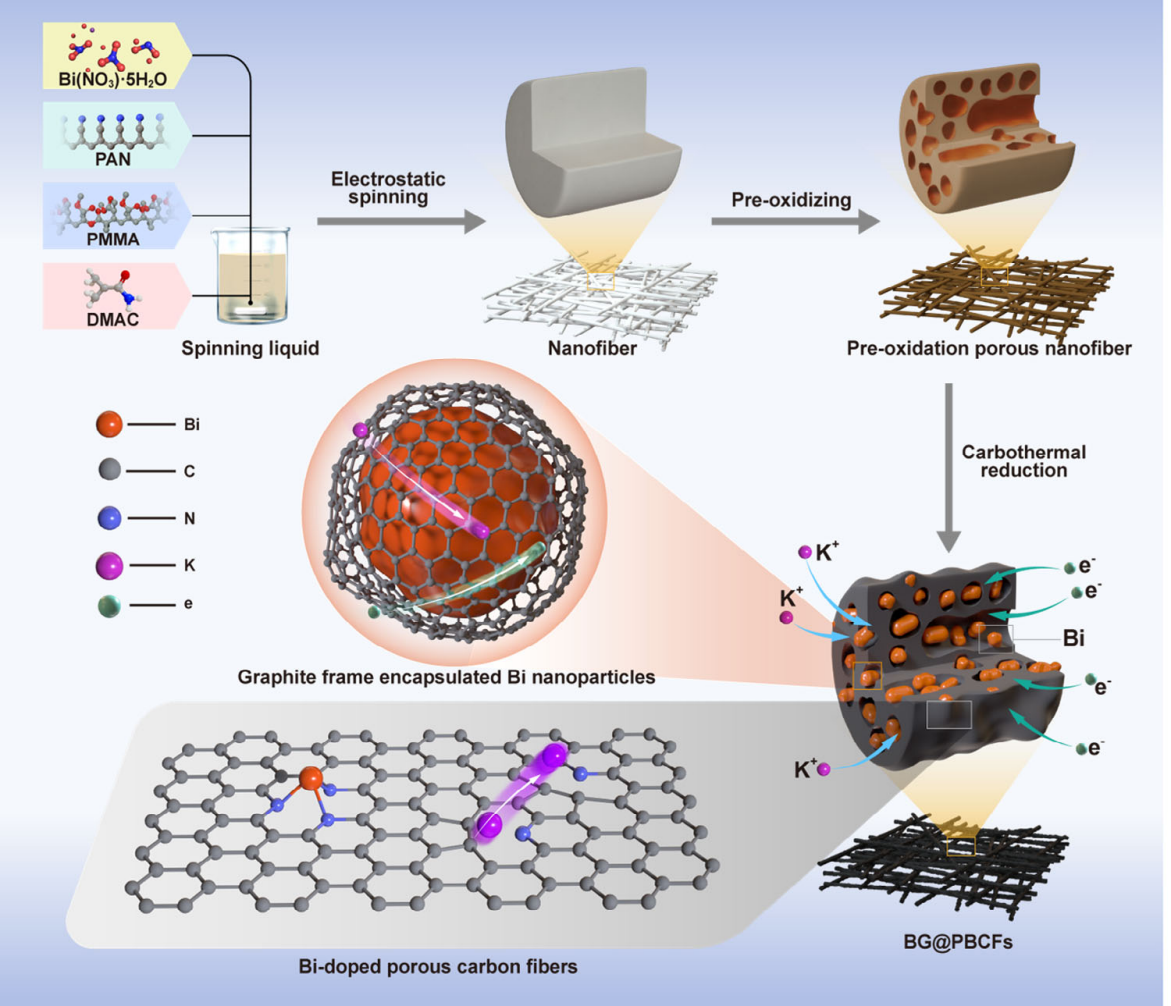
Schematic illustration of the synthesis route of BG@PBCFs.

The synthesis route of the BG@PBCFs materials is illustrated in **Scheme** **1**. First, a uniform spinning solution was prepared by dissolving Bi (NO_3_)_3_·5H_2_O, Polyacrylonitrile (PAN), and Polymethyl Methacrylate (PMMA) in Dimethylacetamide (DMAc). Bi (NO_3_)_3_·5H_2_O was used as the bismuth source, PAN as the carbon source, nitrogen source and reducing agent, and PMMA as the hole‐forming agent. The BG@PBCFs precursor was then obtained by high‐voltage electrostatic spinning, resulting in continuous and uniform nanofibers with a diameter of 400 nm (Figure S1, Supporting Information). Subsequently, the fibers were transferred to a muffle furnace to stabilize the fiber structure and remove the PMMA, resulting in a pre‐oxidation porous nanofiber structure (Figure S2, Supporting Information). Eventually, BG@PBCFs were produced by carbothermal co‐reduction technique under Ar atmosphere (Figure S3, Supporting Information). All the preparation details are given in the experimental section.

**Figure 1:**
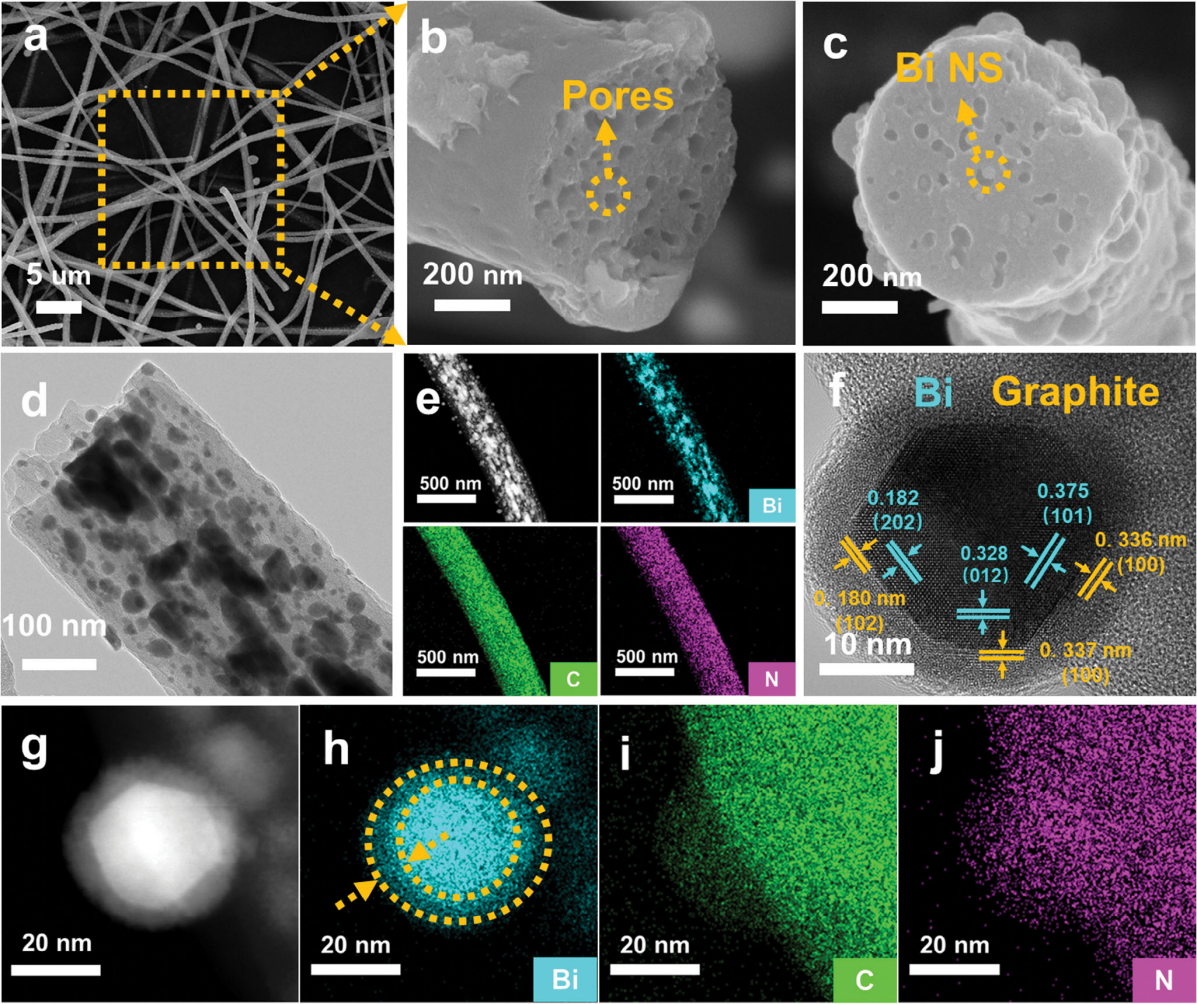
a–c) SEM and d) TEM images of BG@PBCFs, e) Dark Field Scanning Transmission Electron Microscopy (DF‐STEM) image of BG@PBCFs and corresponding elemental mappings for Bi, C, and N elements, f) HRTEM images of BG@ PBCFs, g–j) Elements mapping of f) elements, Bi, C, and N elements.

The microstructure of BG@PBCFs material was observed by SEM and TEM. As shown in **Figure** **1**a, the BG@PBCFs exhibit a continuous fiber morphology with a diameter of ≈400–500 nm. At higher magnification, the internal abundant pore structure was observed on the fiber cross‐section (Figure 1b). Figure 1c reveals that Bi nanoparticles are embedded in pores without completely filling them. This pore structure effectively suppresses the volume expansion of Bi nanoparticles during charge and discharge processes, thus improving their cycling life. Additionally, many Bi nanoparticles with a particle size ranging from 10 to 20 nm are observed inside the fibers (Figure 1d). Energy dispersive spectrometer (EDS) element mapping confirms the uniform distribution of Bi nanoparticles inside the BG@PBCFs fibers and the successful doping of nitrogen elements (Figure 1e). Nitrogen doping enhances the density of active sites, facilitating the adsorption of potassium ions and thereby improving their pseudocapacitive behavior.^[^ ^12^ ^]^ Figure 1f shows the high‐resolution transmission electron microscopy (HRTEM) image of the BG@PBCFs fiber surface, in which the distinct lattice fringe of 0.182, 0.328, and 0.375 nm along three directions correspond to the (012), (202), and (101) crystal planes of bismuth (Bi), respectively (Figures S4–S6, Supporting Information).^[^ ^13^ ^]^ It is worth nothing that Bi nanoparticles are encapsulated by a few layers of graphite, with lattice fringe of 0.180, 0.337, and 0.336 nm corresponding to the (102) and (100) crystal planes of graphite, confirming the presence of few‐layer graphite (Figures S7–S9, Supporting Information).^[^ ^14^ ^]^ The amorphous carbon fiber layers surrounding the Bi nanoparticles are induced by the Bi nanoparticles to form the few‐layer graphite frameworks. This few‐layer graphite framework serves as an efficient interfacial transition that can both accelerate the transfer of K^+^ and electrons and accommodate the volumetric expansion of Bi particles through interlayer sliding, thereby stabilizing the overall structure.^[^ ^15^ ^]^ EDS elemental mapping indicates that a certain number of Bi elements are distributed within the carbon fiber layers, confirming the doping of Bi within the carbon fiber layers (Figure 1g–j). This unique Bi‐doped carbon enables K^+^ to more rapidly penetrate the carbon fiber layers and undergo alloying reactions with the internal few‐layer graphite‐encapsulated Bi nanoparticles. The graphite frameworks induced by Bi and the doping of bismuth elements within the carbon layers collectively enhance the material's overall ion transport capability. Few‐layer graphite frameworks and pore structure enhance the structural stability of the material to mitigate the volume expansion effect of Bi nanoparticles. Additionally, the microstructures of BCFs (Figure S10, Supporting Information) and PCFs (Figures S11 and S12, Supporting Information) were characterized for comparison. BCFs showed a continuous fiber morphology with Bi nanoparticles loaded on the fiber surface but without any pore structure on the cross‐section. Pores were observed on the cross‐section of PCFs fibers, and the TEM images further confirm their internal pore structure.

**Figure 2:**
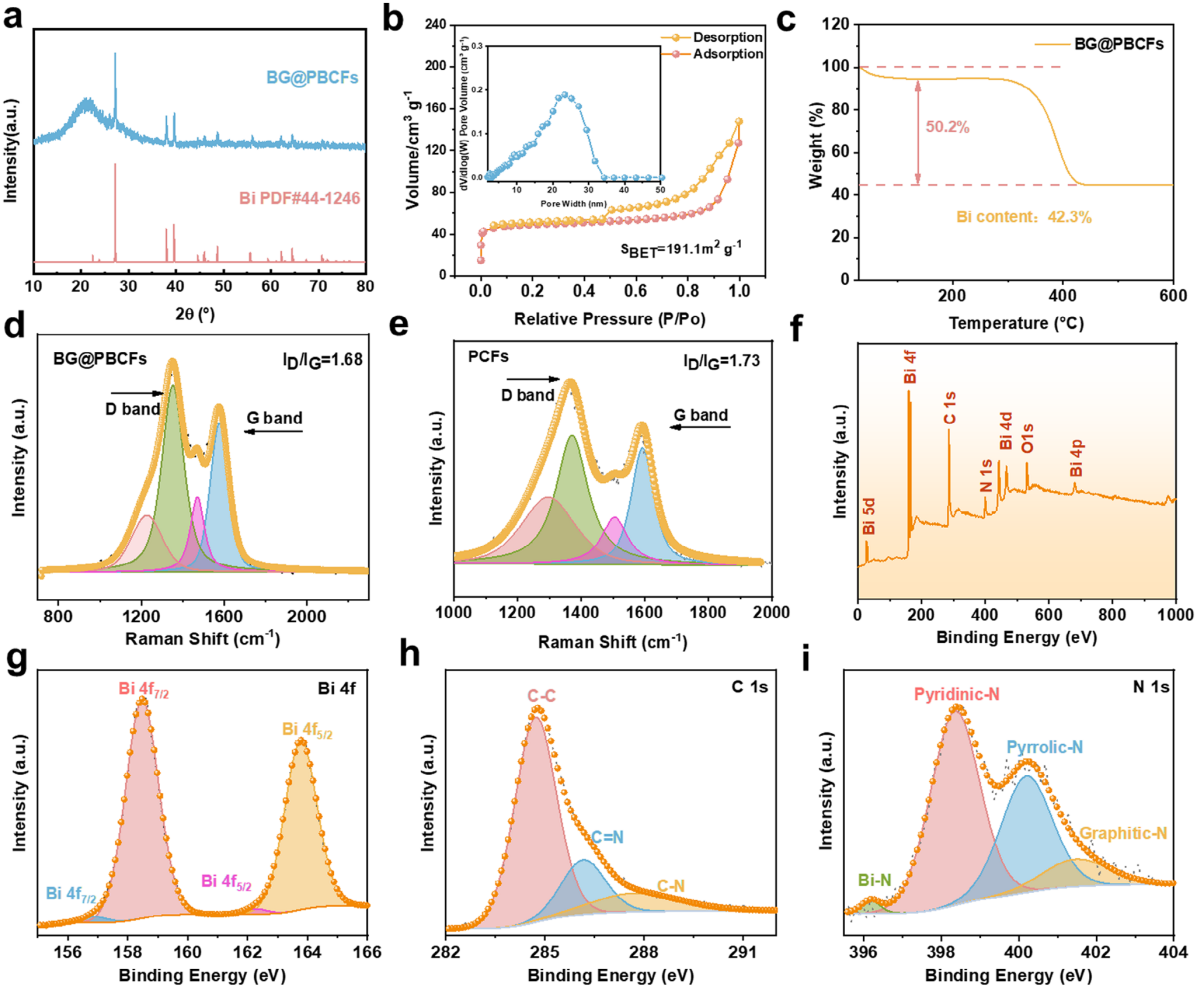
a) XRD patterns of BG@ PBCFs, b) N_2_ adsorption/desorption isotherm, and Pore‐sized distribution of BG@PBCFs, c) TG curve of BG@ PBCFs, d,e) Raman spectrum of BG@PBCFs and PCFs, f–i) XPS spectra of f) survey g) Bi 4f, h) C 1s and i) N1s.

**Figure** **2**a shows the XRD pattern of BG@PBCFs. The peaks at 27.2° and 37.9° are consistent with the typical Bi peak (PDF#44‐1246).^[^ ^13^ ^]^ Both the XRD and HRTEM results fully confirm the presence of Bi. The nitrogen adsorption‐desorption isotherms of BG@PBCFs are shown in Figure 2b. The pore size distribution curve indicates the presence of abundant pore structures in BG@PBCFs, with the majority of pore concentrated in the range of 10–30 nm, consistent with the SEM results. The calculated BET‐specific surface area (SSA) of BG@PBCFs is 191.1 m^2^ g^−1^, which possesses a high surface area that is more accessible to the electrolyte and promotes electrochemical reactions. In contrast, the nitrogen adsorption‐desorption isotherms and the corresponding pore size distribution curve of BCFs are presented in Figure S13 (Supporting Information). BCFs predominantly exhibit a non‐porous structure, with minimal mesoporous and microporous features. The BET surface area of BCFs is only 3.9 m^2^ g^−1^, which is significantly lower than that of BG@PBCFs. The Bi content in BG@PBCFs is determined to be 42.3 wt.% by thermogravimetric analysis (Figure 2c), which is consistent with the results obtained by ICP (Figure S14, Supporting Information). The detailed calculation is based on the following formula:

(1) $$\begin{array}{ccc} \mathrm{Bi}\left(\mathrm{wt}\%\right) & = & \frac{2\;\mathrm{molecular}\;\mathrm{weight}\;\mathrm{of}\;\mathrm{Bi}}{\mathrm{molecular}\;\mathrm{weight}\;\mathrm{of}\;{\mathrm{Bi}}_{2}O_{3}}\\ & & \times \;\frac{\mathrm{final}\;\mathrm{weight}\;\mathrm{of}\;{\mathrm{Bi}}_{2}O_{3}}{\mathrm{initial}\;\mathrm{weight}\;\mathrm{of}\;\mathrm{PBCFs}}\times 100\% \end{array}$$

(2) $$\;=\frac{100\times 2\times 208.98}{465.96}\times \frac{1-0.502-0.0554}{1-0.0554}=42.3\%$$

Raman spectroscopy analysis of BG@PBCFs shows two typical peaks at 1350 and 1574 cm^−1^, attributed to the D and G bands of carbon, respectively, with a corresponding ID/IG value of 1.68 (Figure 2d).^[^ ^16^ ^]^ This confirms the abundance of defects and active sites in BG@PBCFs induced by N and Bi doping, which facilitates the storage of potassium ions in the material. Raman analysis of PCFs reveals an ID/IG ratio of 1.73, which is slightly higher than that of BG@PBCFs. (Figure 2e) This suggests that the few‐layered graphite formed under the induction of Bi particles enhances the overall degree of graphitization in the BG@PBCFs material. The surface properties of BG@PBCFs are investigated using X‐ray photoelectron spectroscopy (XPS), which reveals the presence of C, Bi, N, and O elements in the total spectrum (Figure 2f). The high‐resolution C 1s spectrum displays three peaks at 284.7 eV (C‐C), 286.2 eV (C = N), and 287.7 eV (C‐N) (Figure 2g).^[^ ^11^ ^]^ The high‐resolution XPS spectrum of Bi 4f exhibits two pairs of peaks, corresponding to Bi^0^ (156.9 and 162.3 eV) and the interaction between N and Bi atoms (158.5 and 163.8 eV), respectively (Figure 2h). The stronger Bi‐N peak is mainly attributed to the unique Bi‐doped carbon fiber layers. The weaker Bi^0^ peak is due to the metallic Bi embedded inside the fibers.^[^ ^11^, ^17^ ^]^ This result is consistent with the TEM analysis. The high‐resolution N1s spectrum (Figure 2i) could be fitted into four peaks corresponding to pyridinic‐N (398.4 eV), pyrrolic‐N (400.1 eV), graphitic‐N (401.5 eV), and Bi‐N (396 eV).^[^ ^11^ ^]^ The presence of pyridinic‐N, pyrrolic‐N, and Bi‐N enhances the electrical conductivity of the active sites in the BG@PBCFs, thereby improving the surface pseudocapacitive effect and achieving rapid kinetics.

### 2.2 Electrochemical Properties

**Figure 3:**
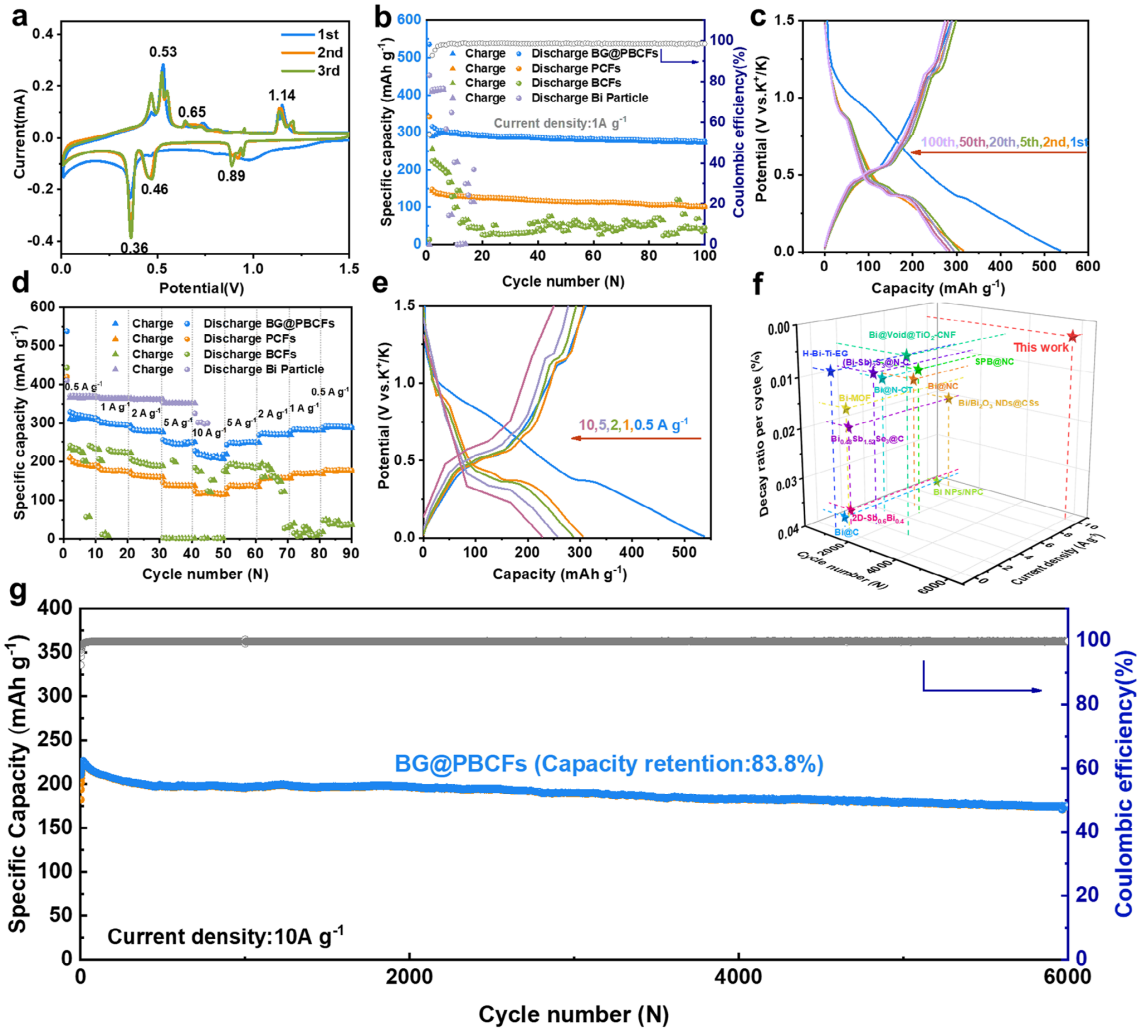
a) First three CV curves of BG@PBCFs at 0.1 mV s^−1^, b) Cycling performance of BG@PBCFs, BCFs, PCFs and Bi particle at 1 A g^−1^, c) GCD curves of BG@ PBCFs at 1 A g^−1^. d) Rate performance of BG@PBCFs, BCFs, PCFs and Bi particle. e) GCD curves tested at various current densities. f) Performance comparisons, g) Long‐cycling performance of BG@PBCFs at 10 A g^−1^.

Using a DME‐based electrolyte, the potassium storage performance of BG@PBCFs nanoparticles was investigated in a half‐cell configuration within a voltage range of 0.1–1.5 V. **Figure** **3**a shows the cyclic voltammetry (CV) curves of the first three cycles of BG@PBCFs at a scan rate of 0.1 mV s^−1^. During the first potassium intercalation process, a broad cathodic peak appeared at 0.9–1.0 V and disappeared subsequently, corresponding to the formation of an irreversible SEI film. The peaks at 0.46 and 0.36 V correspond to the alloying products K_3_Bi_2_ and K_3_Bi, respectively. The three pairs of anodic peaks observed during the subsequent potassium extraction process correspond to the de‐alloying reactions. The peak at 0.53 V corresponds to the process of K_3_Bi de‐potassiation to form K_3_Bi_2_, the peak at 0.7 V corresponds to the process of K_3_Bi_2_ de‐potassiation to form KBi_2_, and the peak at 1.14 V corresponds to the process of KBi_2_ de‐potassiation to form metallic Bi. In the subsequent two cycles, the CV curves show high overlap and cathodic peaks are observed at 0.88, 0.46, and 0.36 V, corresponding to the gradual alloying reaction of Bi‐KBi_2_‐K_3_Bi_2_‐K_3_Bi. Anodic peaks were observed at 0.53, 0.65, and 1.14 V, corresponding to the gradual de‐alloying reaction of K_3_Bi‐K_3_Bi_2_‐KBi_2_‐Bi. These results indicate that BG@PBCFs materials exhibit good electrochemical reversibility and cycling stability. The mass loading of active materials can affect the electrochemical performance of the active material. In this study, we synthesized three different Bi (NO_3_)_3_·5H_2_O addition amounts (1.0, 0.75, and 0.5 g) of BG@PBCFs materials, and the Bi contents in the above materials measured by ICP are 44.7, 33.5, and 22.4 wt.%, respectively (Figure S14, Supporting Information). These images clearly demonstrate that as the concentration of Bi (NO_3_)_3_·5H_2_O increases, the fiber diameter gradually enlarges, and the loading of Bi particles on the fiber surface becomes significantly more pronounced (Figure S15, Supporting Information). The sample with a Bi content of 44.7 wt.% exhibits the best battery performance, with a capacity of 318 mAh g^−1^ at 0.5 A g^−1^, while the capacities of the samples with Bi contents of 33.5 and 22.4 wt.% are 259 and 228 mAh g^−1^, respectively, under the same condition (Figure S16, Supporting Information). These results indicate that increasing the mass loading of the active material is beneficial to the contribution of capacity. However, excessive addition of Bi (NO_3_)_3_·5H_2_O can affect the formation of fibers. We also configured electrostatic spinning solutions with higher concentrations of (10, 15, and 20 wt.%), and the experimental observations revealed a critical solubility threshold. When the Bi (NO_3_)_3_·5H_2_O content exceeded 10 wt.%, incomplete dissolution occurred in DMAc solvent, as evidenced by the turbid appearance of the solution (Figure S17, Supporting Information). This phase instability fundamentally compromised the homogeneity of the spinning solution. Subsequent electrospinning at high voltage with 15 and 20 wt.% formulations yielded distinct morphological outcomes. SEM analysis (Figure S18, Supporting Information) of the 15 wt.% samples exhibited fibrous structures with surface irregularities, including discrete particulate deposits and microscale spherical droplet formations. Notably, the 20 wt.% formulation failed to produce continuous fibers, instead resulting in sporadic solution droplets on the collector. This phenomenon is attributed to partial needle clogging caused by the heterogeneous nature of the solution, which disrupted the stable jet formation during the electrohydrodynamic process. Therefore, Bi (NO_3_)_3_·5H_2_O additional amount of 1 g was chosen for subsequent studies. To determine the optimal synthesis process, we synthesized BG@PBCFs materials with three different PAN‐PMMA ratios (1.0–0.2, 0.8–0.4, and 0.6–0.6). The sample with a PAN‐PMMA ratio of 0.8–0.4 exhibits the best battery performance (Figure S19, Supporting Information). The sample with a PAN‐PMMA ratio of 1.0–0.2 shows a capacity of only 229 mAh g^−1^ at 0.5 A g^−1^, as some of the metallic Bi particles are wrapped by fibers and do not participate in the reaction. Although the sample with a PAN‐PMMA ratio of 0.6–0.6 provided a specific capacity of 311 mAh g^−1^ at 0.5 A g^−1^, it quickly decays due to the instability of the final fiber structure caused by excessive pore‐forming agent addition, which collapses easily during the reaction. Therefore, BG@PBCFs with a PAN‐PMMA ratio of 0.8‐0.4 are selected. The effects of annealing temperature and duration on the synthesis and properties of BG@PBCFs were systematically investigated. The designation of BG@PBCFs samples prepared under different annealing conditions are provided in Table S1 (Supporting Information). XRD analysis revealed that samples annealed at 600 °C for 1 and 2 h exhibited the most prominent Bi diffraction peaks (Figure S20, Supporting Information). This can be attributed to the fact that metallic Bi begins to sublimate at elevated temperatures, leading to a reduction in Bi content within the samples. In contrast, at 500 °C, the carbothermal reduction process was incomplete, resulting in insufficient reduction of Bi nanoparticles and consequently weaker diffraction peak intensities for BG@PBCFs‐500 compared to BG@PBCFs‐600 and BG@PBCFs‐600‐2h. SEM analysis further corroborated these findings (Figure S21, Supporting Information). Compared to the BG@PBCFs‐500 and BG@PBCFs‐600‐2 h, the fiber surfaces of BG@PBCFs‐700 to BG@PBCFs‐1000 appeared nearly smooth due to the sublimation of Bi particles, which were carried away by the gas flow. The reduced Bi content also implies a lower capacity contribution. Further rate capability studies on these samples yielded results consistent with the aforementioned results (Figure S22, Supporting Information). Among the samples, BG@PBCFs‐600‐2 h exhibited the highest capacity contribution. However, its prolonged annealing time led to the agglomeration and growth of Bi particles, which negatively the structural stability. Therefore, considering both capacity and stability, an annealing temperature of 600 °C for 1 h was determined to be the optimal condition.

Figure 3b indicates the cyclic performance of BG@PBCFs, BCFs, PCFs, and BP at a current density of 1 A g^−1^. BG@PBCFs exhibit the best cyclic performance, maintaining a high reversible capacity of 277 mAh g^−1^ after 100 cycles. In comparison, BCFs and PCFs perform poorly. BCFs can provide a reversible capacity of 256 mAh g^−1^ at the beginning of the cycle, but it quickly decays due to the lack of pore structure characteristics, which cannot buffer the volume expansion during alloying, leading to the deterioration of the overall fiber structure. PCFs have a porous fiber structure and exhibit stable cyclic performance, but their main capacity contribution comes from the carbon material, resulting in a low capacity. Commercial BP‐based anodes exhibit a higher Bi content compared to BG@PBCFs‐based anodes, which endows them a higher initial capacity. However, the absence of few‐layered graphite, which facilitates rapid potassium‐ion conduction, and the lack of a Bi‐doped porous carbon nanofiber structure that stabilizes the architecture, result in inferior cycling performance. Figure 3c shows the galvanostatic charge/discharge (GCD) curves of BG@PBCFs at different cycles under 1 A g^−1^, and the GCD curves have obvious plateaus and good curve matching between different cycles, indicating excellent cyclic stability and reversibility of BG@PBCFs. Figure 3d compares the rate performance of BG@PBCFs, BCFs, PCFs, and BP in the range of 0.5–10 A g^−1^. The results show that BG@PBCFs have the best rate performance, with average capacities of 318, 294, 281, 248, and 215 mAh g^−1^ at 0.5, 1, 2, 5, and 10 A g^−1^, respectively. The initial coulombic efficiency of BG@PBCFs at a current density of 0.5 A g⁻¹ is 58%. When the current density is restored to 0.5 A g^−1^, the capacity can recover and remain stable in subsequent cycles, with a capacity retention rate of 91%. BCFs exhibit poor rate performance and cannot provide stable capacity when the current density is restored to 0.5 A g^−1^. PCFs have average capacities of 194.5, 175.6, 160.0, 136.5, and 118.3 mAh g^−1^ at 0.5, 1, 2, 5, and 10 A g^−1^, respectively, with an overall low capacity. When the current density is restored to 0.5 A g^−1^, the capacity retention rate is 91%. Commercial BP‐based anodes exhibited a precipitous decline in performance at high current densities, resulting in battery failure. This observation further corroborates the significance of the few‐layered graphite framework and the Bi‐doped porous carbon nanofiber structure in enhancing the electrochemical stability and performance of the electrode. In addition, even at 10 A g^−1^, BG@PBCFs still have obvious charge/discharge plateaus (Figure 3e), indicating their extremely fast kinetic process. To elucidate the mechanism of the excellent high‐rate performance of BG@PBCFs, the contributions of capacitance and diffusion to the battery capacity were analyzed. The results show that the capacity is mainly controlled by capacitance, and the proportion of capacitance contribution gradually increases from 79.3% to 98.5% with the increase of scanning rate (Figures S23 and S24, Supporting Information). This high capacitance contribution endows additional capacity and ultra‐fast electron/ion transfer kinetics. To mitigate the potential influence of sp2‐sp2 interaction domains within the porous graphite framework, which may hinder the efficient ion transport, we performed Galvanostatic Intermittent Titration Technique (GITT) tests on BG@PBCFs and BCFs to accurately evaluate the diffusion rates of potassium ions. Figures S25–27 (Supporting Information) illustrate the GITT profiles as a function of specific capacity, with the corresponding potassium diffusion coefficients (D_K⁺_) calculated using Equation (3).^[^ ^16^ ^]^ The results demonstrate that BG@PBCFs, with a mesoporous few‐layered graphite framework, exhibit superior potassium‐ion transport rates (charging: 10^−6^–10^−8^ cm^2^ s^−1^; discharging: 10^−6^–10^−10^ cm^2^ s^−1^) compared to BCFs (charging: 10^−7^–10^−9^ cm^2^ s^−1^; discharging: 10^−8^–10^−11^ cm^2^ s^−1^). These findings confirm that the few‐layered graphite framework facilitates rapid mobility of potassium‐ion. BG@PBCFs exhibit impressive long‐term cyclic performance, providing a discharge‐specific capacity of 160 mAh g^−1^ after 6000 cycles at a current density of 10 A g^−1^, with a capacity retention rate of 83.8%. The average attenuation rate per cycle is 0.00278%, which is better than the recently reported Bi‐based anode materials (Figure 3g,f and Table S2, Supporting Information). The excellent long‐term cyclic stability is mainly attributed to the design of the unique few‐layer graphite frameworks and Bi‐doped porous carbon fiber structure.

(3) $$D_{K^{+}}=\frac{4}{\pi \tau }\;{\left(\frac{n_{m}V_{m}}{S}\right)}^{2}{\left(\frac{\Delta E_{s}}{\Delta E_{t}}\right)}^{2}$$

$D_{K^{+}}$: potassium diffusion coefficients τ: Relaxation time

*n_m_*: Number of moles per unit volume *V_m_*: Molar volume

*S*: Surface area of the electrode Δ*E_s_*: Pulse‐induced voltage changes

Δ*E_t_*: Voltage change for constant current charging (discharging)

### 2.3 Potassium Storage Mechanism

**Figure 4:**
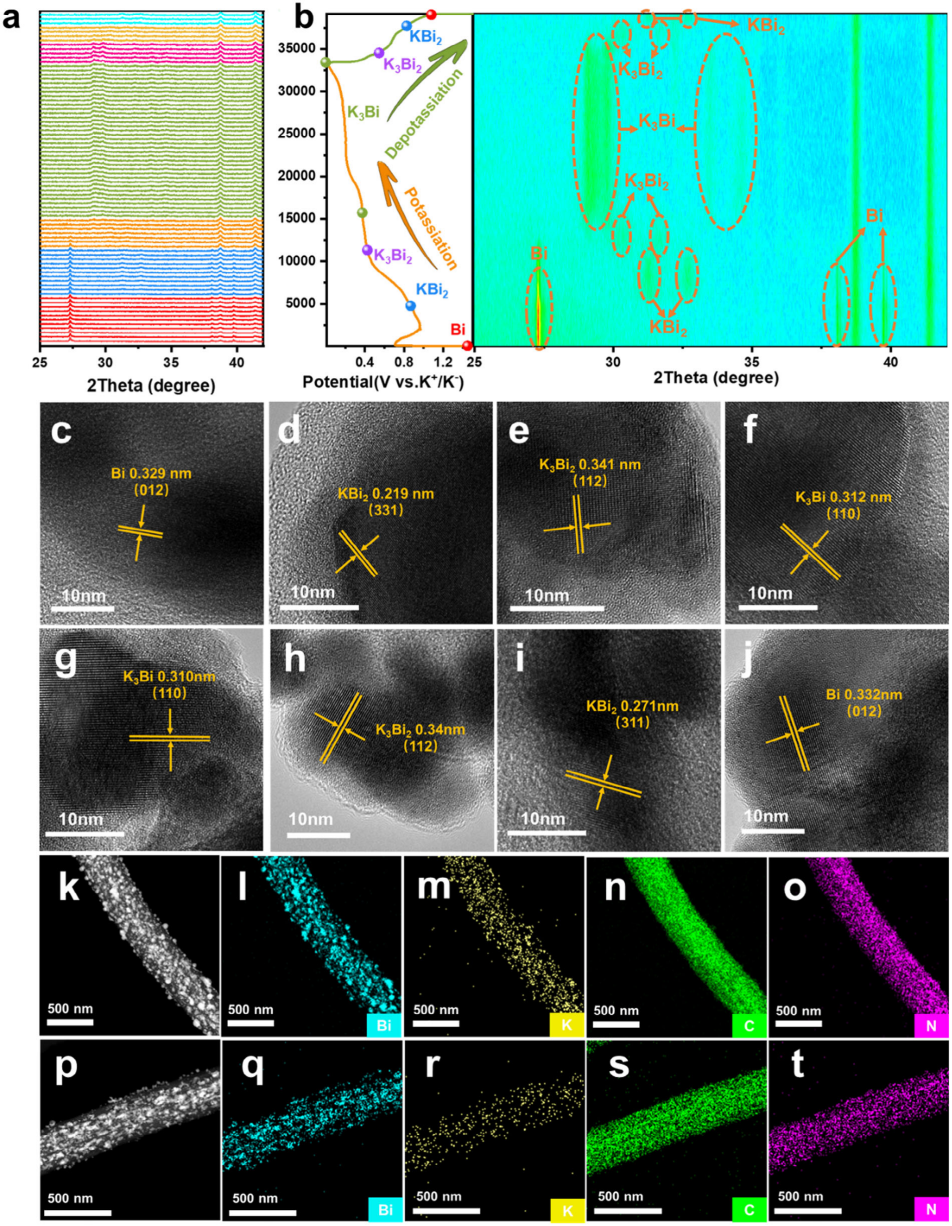
a,b) In situ XRD. c–g) Ex situ HRTEM images after discharging to 1.5, 0.85, 0.42, 0.36, and 0.01 V, respectively. h–j) Ex situ HRTEM images after charging to 0.55, 0.75, and 1.2 V, respectively. k) DF‐STEM image of BG@PBCFs after fully potassiation. The corresponding elemental mappings of i) Bi, m) K, n) C, and o) N elements. p) DF‐STEM image of BG@PBCFs after fully depotassiation. The corresponding elemental mappings of q) Bi, r) K, s) C, and t) N elements.

The reaction mechanism of BG@PBCFs composite material during cycling is revealed using in‐situ XRD testing, and the corresponding XRD spectra are shown in **Figure** **4**a,b. During the first potassium intercalation process, three peaks of metallic Bi are detected at 27.2°, 37.9°, and 39.6°, corresponding to PDF#44‐1246. As the potassium intercalation proceeds, the intensity of the Bi peak weakens and disappears, and a distinct diffraction peak corresponding to the preliminary alloying product KBi_2_ (PDF#03‐0698) appears at 31.2° and 32.6° when the discharge voltage reaches 0.86 V.^[^ ^18^ ^]^ With further potassium ion intercalation, KBi_2_ continues to undergo alloying reaction, and a new diffraction peak corresponding to the intermediate alloying product K_3_Bi_2_ appears at 30.3° and 31.7° when the voltage drops to 0.46 V. As the discharge process continues, the diffraction peaks of the final alloying product K_3_Bi appear at 28.8°, 29.5°, 33.2°, and 34.4°. The subsequent stepwise charging process involves the de‐alloying of K_3_Bi to K_3_Bi_2_, followed by further de‐alloying of K_3_Bi_2_ to KBi_2_, and finally the complete de‐alloying to Bi metal. However, in the in situ XRD analysis, the Bi diffraction peaks were not observed in Figure 4b. This may be attributed to the evaporation of the DME electrolyte within the in situ XRD cell, which is equipped with a beryllium window.^[^ ^19^ ^]^ The evaporation of the DME electrolyte inferred with the de‐intercalation of potassium ions during the measurement, thus preventing the formation of the expected charging plateau at 1.14 V. As a result, the de‐alloying reaction from KBi₂ to Bi was not fully realized, and as a result, the Bi diffraction peaks were not detected. Non‐in situ HRTEM and non‐in situ TEM element mapping are further used to analyze the K storage mechanism. Bi, KBi_2_, K_3_Bi_2_, and K_3_Bi are detected at discharge potentials of 1.5, 0.85, 0.42, 0.36, and 0.01 V, respectively (Figure 4c–g). K_3_Bi_2_, KBi_2_, and Bi are detected at charging potentials of 0.55, 0.75, and 1.2 V (Figure 4h–j), consistent with the in‐situ XRD results. In addition, the uniform mapping of K and Bi elements in the complete potassiumized spectrum (Figure 4k–o) is attributed to the formation of K_3_Bi. The weak intensity of the K element after complete de‐potassiation (Figure 4p–t) indicates complete de‐alloying reaction, which can be completely de‐alloyed to Bi metal, further proving the good reversibility of BG@PBCFs. The weak intensity of the potassium element mapping is mainly attributed to the residual K^+^ in the SEI film. Additionally, the anodic peaks near 1.14 V in the CV curves (Figure 3a), as well as the charging plateau near 1.14 V in the charge‐discharge profiles, provide further evidence for the KBi₂ → Bi reaction step. Combining in‐situ XRD with non‐in situ HRTEM and non‐in situ TEM analysis, the entire electrochemical process and related mechanisms of BG@PBCFs are briefly summarized as follows.

Intercalation process of potassium ion:

(4) $$2Bi+K^{+}+e^{-}\to KBi_{2}$$

(5) $$KBi_{2}+2K^{+}+2e^{-}\to K_{3}Bi_{2}$$

(6) $$K_{3}Bi_{2}+3K^{+}+3e^{-}\to 2K_{3}Bi$$

De‐intercalation process of potassium ion

(7) $$2K_{3}Bi\to K_{3}Bi_{2}+3K^{+}+3e^{-}$$

(8) $$K_{3}Bi_{2}\to KBi_{2}+2K^{+}+2e^{-}$$

(9) $$KBi_{2}\to 2Bi+K^{+}+e^{-}$$

### 2.4 Kinetic Mechanism

**Figure 5:**
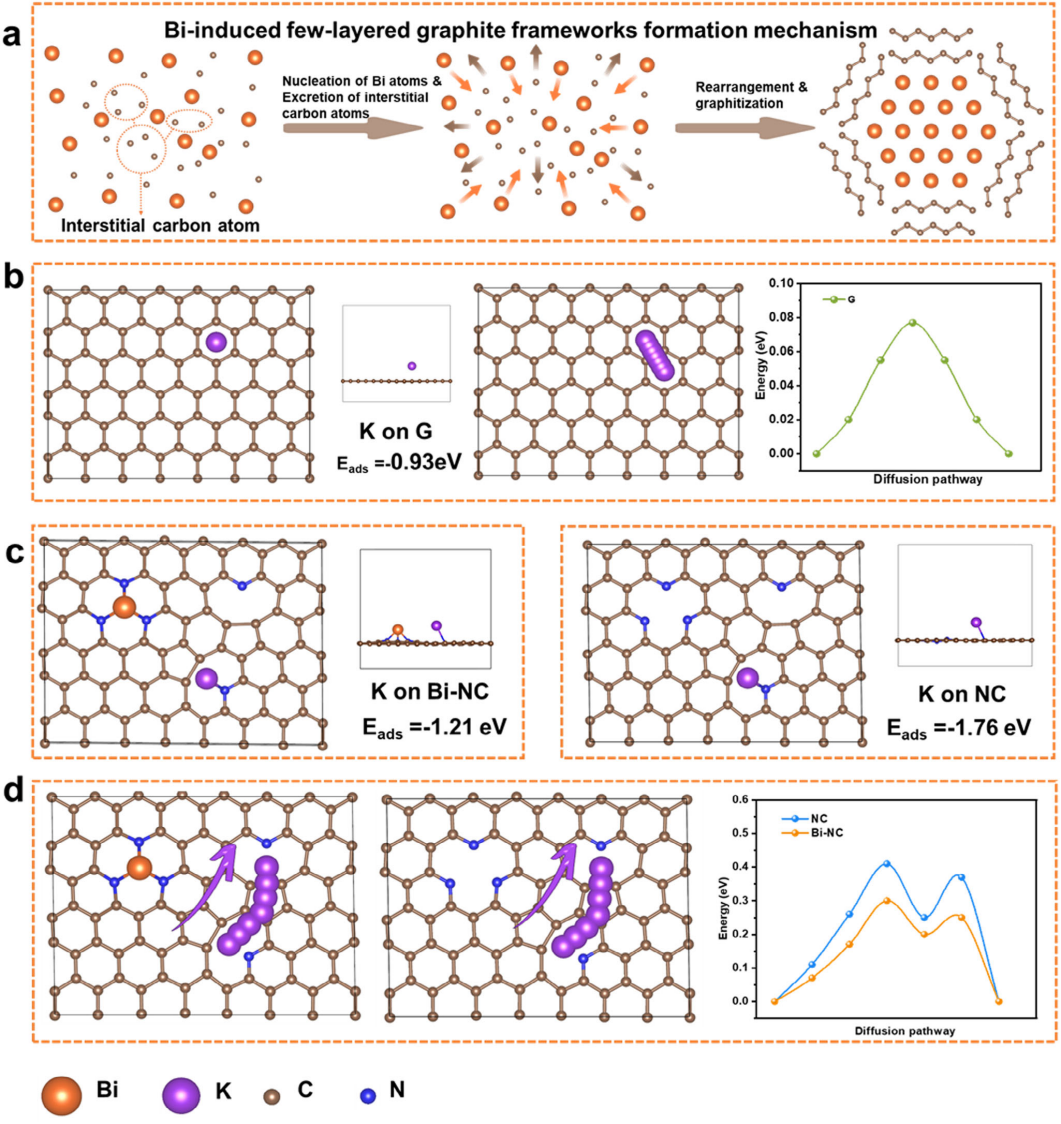
a) Schematic diagram of Bi‐induced few‐layered graphite formation; b–d) Adsorption models, energies, migration paths, and energy barriers of K on b) graphite; c) Bi‐NC; d) NC. (Orange ball: Bi, Purple ball: K, Blue ball: N, Brown ball: C).

**Figure** **5**a illustrates the schematic diagram of the formation process of few‐layered graphite induced by Bi. The process adheres to the graphitization mechanism involving the rearrangement of interstitial carbon atoms during the nucleation of bismuth. Under elevated temperature conditions, a minor fraction of amorphous carbon dissolves and diffuse into the molten Bi, where interstitial carbon atoms are present among the Bi atoms. During the nucleation process as Bi atoms aggregate, the internal interstitial carbon atoms among Bi atoms are expelled at the same time. The process is analogous to the graphitization mechanism of solid‐state homogeneous catalysis proposed by Robert D. Hunter et al., wherein carbon atoms diffuse into the interstitial sites of the iron lattice.^[^ ^20^ ^]^ Owing to the free enthalpy difference between amorphous carbon and graphite carbon, these expelled interstitial carbon atoms precipitate at the Bi‐C interface in the form of graphite carbon, leading to the formation of few‐layered graphite, with the exterior remaining encapsulated by amorphous carbon (Figure 1f).^[^ ^21^ ^]^ Density functional theory (DFT) computational results demonstrate that nitrogen atoms doped within amorphous carbon are more readily incorporated with Bi atoms during the graphitization process, a finding that corroborates the results obtained from XPS (Figures S28 and S29, Supporting Information).

To elucidate the kinetic mechanism of K^+^ in BG@PBCFs, we further investigate the process by which K^+^ traverse the Bi‐doped carbon fiber layers and the graphite frameworks to undergo alloying reactions with the internal Bi nanoparticles. Models of K on G and B‐NC are constructed to represent the graphite frameworks and Bi‐doped carbon fiber layers (Figure 5b,c). A K on NC model is created to simulate the PCFs carbon layer, and two carbon rings containing irregular N6 are used to simulate the defective N‐doped carbon layer for comparison (Figure 5c). The adsorption energies of K^+^ on the three models are calculated; the adsorption energy of K^+^ on G (−0.93 eV) and B‐NC (−1.21 eV) are both lower than that on NC (−1.76 eV), with the lowest on G. This favors the migration of K^+^ on the graphite frameworks and Bi‐doped carbon fiber layers. The migration barriers for K^+^ in the three models are further calculated, with the migration paths viewed from Figure 5b,d, taking the movement of through a six‐membered carbon ring as an example. According to the results of the migration barriers, the diffusion of K^+^ in the graphite frameworks, Bi‐doped carbon fiber layers, and N‐doped carbon fiber layers exhibit differences. During the traversal of the carbon rings, K on the graphite frameworks shows the lowest energy barrier, indicating that the graphite frameworks can serve as an efficient transition interface to accelerate the transfer of K^+^, making the ion distribution on the surface of Bi nanoparticles more uniform and generating high‐rate capability. Compared with NC, K on B‐NC have smaller migration barriers throughout the entire migration path. The doping of Bi in the carbon fiber layer allows K^+^ to diffuse rapidly within the carbon layer and undergo alloying reactions with the internal Bi nanoparticles, thereby enhancing the electrochemical reaction activity and generating high‐rate capability. The above analysis indicates that the graphite frameworks and Bi‐doped carbon fiber can improve the diffusion kinetics of K^+^ in BG@PBCFs. It can be inferred that BG@PBCFs exhibit high‐rate capability during the alloying process and maintain the stability of the electrode, which is consistent with the experimental results. Figure S30 (Supporting Information) further elucidates the transport mechanism of potassium ions within individual Bi nanoparticles. Under the application of external voltage, potassium ions uniformly diffuse into the Bi nanoparticles, undergoing a progressive alloying reaction from the outer surface to the inner core until the completion of the potassium insertion process. This behavior is consistent with the mechanism of potassium insertion into nanostructured Bi proposed by Zhang et al.^[^ ^22^ ^]^ The nanoscale Bi particles follow a stepwise solid‐solution pathway, characterized by rapid ionic transport kinetics and uniform diffusion.

### 2.5 Structure Stability Mechanism

**Figure 6:**
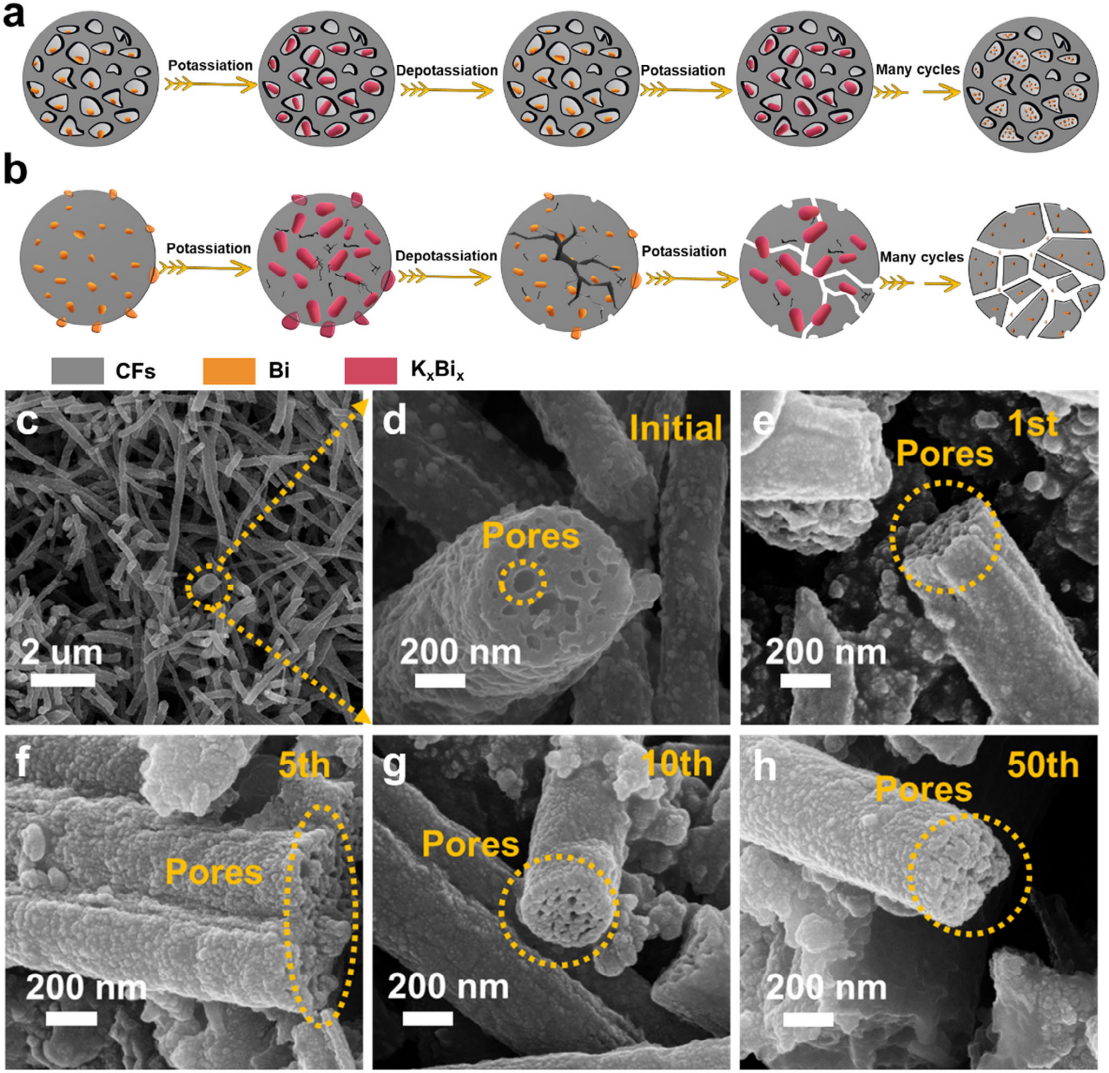
Schematic illustrations for structural collapse mechanism (side cross‐section view) of the electrodes BG@PBCFs a) and BCFs b) upon many cycles. c–f) Ex situ SEM images of BG@PBCFs after different cycles at 10A g^−1.^

The excellent long‐term cycling performance of BG@PBCF_S_ electrode material can be attributed to several factors. First, the few‐layered graphite frameworks with rapid K^+^ transport capabilities are encapsulated around Bi particles, which can effectively suppress the volume expansion of Bi particles at the microstructural level through the sliding process between adjacent graphite layers. Second, the design of the porous structure provides an effective buffering space for Bi nanoparticles, maintaining the structural integrity of the electrode material during the alloying process (**Figure** **6**a). In contrast, BCFs without a porous structure (Figure 6b) undergo significant volume changes of Bi particles during cycling, leading to fiber structure damage, Bi particle fragmentation and detachment, and poor electrochemical activity, as shown in the simulation graph. To further verify the above analysis, ex situ SEM analysis was performed on BG@PBCFs and BCFs after different cycling stages at a current density of 10 A g^−1^ (Figure 6c–h, Figure S31, Supporting Information). With continued cycling, BG@PBCFs can still preserve their fiber structure, and the pore structure is not filled, indicating that the porous design could effectively accommodated the volume expansion of Bi particles. In contrast, during the initial 1–10 cycles, the BCFs exhibited progressive swelling, followed by the formation cracks and eventual fragmentation. After 50 cycles, the fibrous morphology of the BCFs is almost entirely lost, remaining only fragmented residues. These observations provide direct experimental evidence for the inferior cycling stability of non‐porous BCFs. The Nyquist curve of the electrochemical impedance spectroscopy (EIS) at different cycles (Figure S32, Supporting Information) shows that the contact resistance and charge transfer resistance of BG@PBCFs are low and stable, consistent with the electrochemical performance results. Third, the 3D porous morphology structure formed by the stacking of fibers can increase the available electrode‐electrolyte contact interface, shorten the diffusion channel of K^+^, and promote the entry of electrolyte into the electrode. Fourthly, Bi metal nanoparticles embedded in porous carbon fibers can inhibit the aggregation of Bi metal, improve the utilization efficiency of active materials, and thus improve the capacity and cycling stability. The above analysis indicates that the dual carbon layers (few‐layered graphite and Bi‐doped porous carbon fiber layers) combined with a rational structural design can significantly enhance the structural stability of BG@PBCFs. Benefiting from these advantages, the BG@PBCFs exhibit excellent long‐term cycling performance, consistent with experimental results.

### 2.6 PTCDA|| BG@PBCFs Full Cell Performance

**Figure 7:**
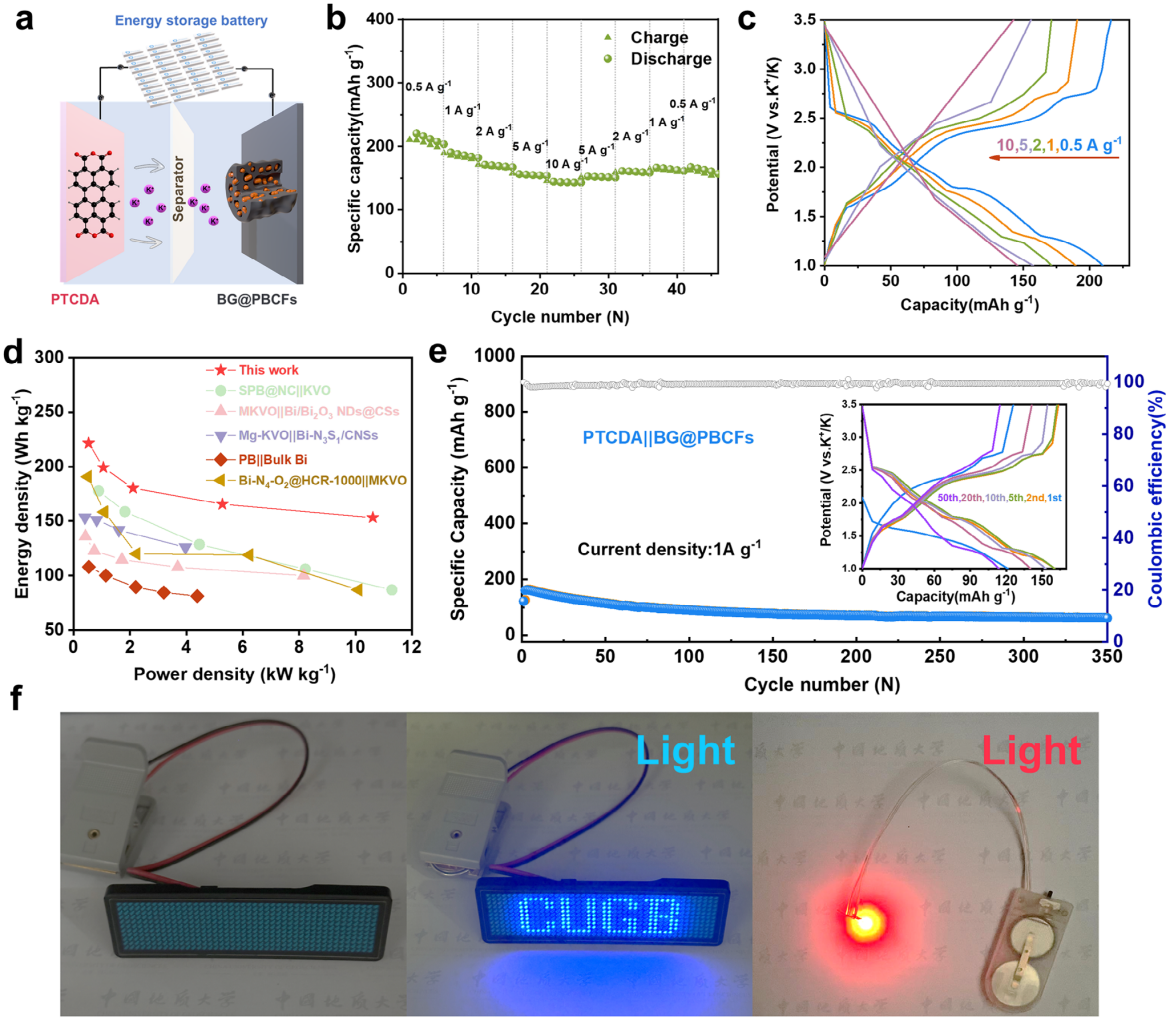
a) Schematic illustration of PTCDA||BG@ PBCFs full cell; b)Rate performance; c) GCD curves tested at various current densities; d) Ragone plot. e) Cycling performance at 1 A g^−1^and GCD curves of PTCDA||BG@ PBCFs at 1 A g^−1^; f) LED badge lighted up by PTCDA||BG@PBCFs full cell.

The study demonstrates that BG@PBCFs exhibit excellent half‐cell performance. To explore their practical application, a full PIB was assembled with PTCDA as the cathode and BG@PBCFs as the anode (**Figure** **7**a). Commercial PTCDA was selected and annealed at 400 °C to enhance its conductivity before use, and its cycling performance was tested at a current density of 1 A g^−1^ (Figure S33, Supporting Information). The BG@PBCFs anode was cycled 5 times at 0.5 A g^−1^ and finally pre‐potassiated by discharging to 0.1 V. The capacity of the full cell was calculated based on the mass of the anode active material. The full cell obtained specific capacities of 206, 184, 169, 155, and 144 mAh g^−1^ at current densities of 0.5, 1, 2, 5, and 10 A g^−1^, respectively (Figure 7b), with the corresponding GCD curves shown in Figure 7c. Notably, the PTCDA||BG@PBCFs full cell exhibited excellent energy density (E) and power density (P). Based on the mass of the cathode and anode, the full cell achieved a maximum energy density of 221 Wh kg^−1^ at a power density of 527.9 W kg^−1^ within the voltage range of 1–3.5 V. Moreover, the energy density remained stable at various current densities without significant sharp degradation, sustaining an energy density of 165.6 Wh kg^−1^ even at a high power density of 5274.9 W kg^−1^. This performance surpasses the majority of Bi‐based potassium‐ion full cells reported in the literature (Figure 7d and Table S3, Supporting Information).^[^ ^10^, ^16^, ^18^, ^23^ ^]^ The cycling performance at 1 A g^−1^ of PTCDA||BG@PBCFs full cell and the corresponding GCD curves are shown in Figure 7e, the first discharge capacity of the PTCDA||BG@PBCFs full cell at 1A g^−1^ was 122 mAh g^−1^. After the initial capacity fade, the PTCDA||BG@PBCFs full cell exhibited remarkable stability, with a capacity retention rate of over 80% in the subsequent 200 cycles. The primary reason for the initial capacity loss is primarily attributed to the relatively limited cycling stability of PTCDA. Under the same current density, PTCDA experiences a significant capacity degradation within the first 150 cycles, which is consistent with the trend observed in our assembled full cell. We are confident that replacing PTCDA with a more robust cathode material in future studies would result in superior long‐term cycling performance for the BG@PBCFs anode in full cells, which will be a key focus in our ongoing research. The full cell could easily power a blue “CUGB” (abbreviation of “China University of Geoscience (Beijing)”) LED badge with significant brightness and light up a red LED bulb, demonstrating its application potential (Figure 7f). Although it performed well in the full PIB, further optimization and improvement of capacity, cycle life, and energy/power density are expected to be achieved by adjusting the process of the cathode material and the mass ratio of the electrode materials.

## 3 Conclusion

In this study, we have synthesized a few‐layered graphite encapsulated Bi nanoparticles embedded in Bi‐doped porous carbon fibers composite. In this structure, Bi serves as a doping element incorporated into the porous carbon fiber matrix, and simultaneously, Bi atoms nucleate to form a few‐layered graphite encapsulated Bi nanoparticles, which are embedded within the fiber channels as active materials for electrode applications Experimental studies and theoretical calculations demonstrate that the few‐layered graphite frameworks induced by Bi acts as an efficient transition interface that effectively promotes charge transfer and diffusion. The Bi‐doped porous carbon fiber layer further facilitates rapid K^+^ diffusion within the carbon layers. The design strategy of dual carbon layers with porous structure greatly enhances the structural stability of BG@PBCFs anode during the cycling process. The multifunctional nature of Bi‐metal and the structural design of dual carbon layers lead to the outstanding high‐rate performance and ultralong cycling stability of BG@PBCFs (215mAh g^−1^ at 10 A g^−1^, a capacity retention rate of 83.8% after more than 6000 cycles at 10 A g^−1^ and an ultra‐low decay rate of 0.00278% per cycle.) The assembled PTCDA|| BG@PBCFs full cell also exhibits excellent rate performance, with a capacity of 144 mAh g^−1^ at 10 A g^−1^. This full cell can achieve a maximum energy density of 221 Wh kg^−1^ at 527.9 W kg^−1^, showing practical application potential. Through electrochemical testing and DFT calculations, its fast charge/ion transfer kinetics characteristics are determined. Combining in situ XRD, non‐in situ TEM, and non‐in situ SEM reveal reveals the K^+^ storage mechanism and structure stability mechanism of BG@PBCFs. This work would pave a new path for developing high‐performance anode materials to address the increasing demand for energy storage.

## 4 Experimental Section

### Synthesis of BG@PBCFs

BG@PBCFs precursors were prepared by the electrospinning method. Typically, 1 g of Bi (NO_3_)_3_·5H_2_O (Xilong Chemical Co.), 0.8 g of Polyacrylonitrile (PAN, Average Mw = 150000, Aladdin Reagent Co.), and 0.4 g of Polymethyl Methacrylate (PMMA, Aladdin Reagent Co.) were dissolved in 7.8 g of Dimethylacetamide (DMAc 99.0%, Aladdin Reagent Co.) and stirred at 60 °C for 6 h until the solution clarified. The resulting solution was then injected into a 5 mL syringe with a 23G pinhole aperture at an injection speed of 0.8 mL min^−1^. The needle was connected to the cathode of the high‐voltage power supply equipment. The voltage is set to 15 kV, and the negative pressure of the metal drum collection equipment is 2 kV. The distance between the needle and the metal collector is 15 cm. The fibers were collected, dried at 60 °C for 6 h, and then heated in a muffle furnace at 280 °C for 1 h with a heating rate of 1 °C min^−1^. Subsequently, the product was annealed in Ar atmosphere at 600 °C for 1 h with a heating rate of 2 °C min^−1^ to obtain BG@PBCFs. The same conditions were used to prepare Porous carbon nanofibers (PCFs, without Bi (NO_3_)_3_·5H_2_O) and Bi‐loaded carbon nanofibers (BCFs, without PMMA). Additionally, two more BG@PBCFs with varying Bi contents were synthesized. The mass of Bi (NO_3_)_3_·5H_2_O was adjusted to 0.75 and 0.5 g, respectively, while keeping all other preparation conditions constant. Two other BG@PBCFs with different PAN and PMMA additions were also synthesized with (1.0 g PAN and 0.2 g PMMA) and (0.6 g PAN and 0.6 g PMMA), respectively, and other synthetic conditions were the same. BG@PBCFs were synthesized under various annealing conditions, including different temperatures (500, 700, 800, 900, and 1000 °C) and a specific condition of 600 °C for 2 h. Bismuth particles (BP, 99.5%) were purchased from McLean & Co. The phase and morphological characteristics of these particles are shown in Figures S34 and S35 (Supporting Information). The commercial PTCDA (98%, Aladdin Reagent Co.) was annealed in Ar atmosphere at 450 °C for 3 h with a heating rate of 5 °C min^−1^.

### Materials Characterization

The phase composition was measured by power X‐ray diffraction (Bruker, D8 Advance) in the range 2θ = 10°–80°. The chemical contents were detected by inductively coupled plasma‐optical emission spectrometry (ICP‐OES, Agilent 7800 MS). The structure and morphology of all samples were observed by a JOEL JSM 7001F field emission scanning electron microscopy (SEM) with an energy‐dispersive X‐ray spectrometer (EDS) and an FEI Tecnai G2 F30 transmission electron microscopy (TEM). The element valence states were tested by X‐ray photoelectron spectroscopy (XPS, Thermo Scientific K‐Alpha). Specific surface area and pore size were tested using nitrogen adsorption and desorption. (BET, Micromeritics ASAP 2460).TGA was performed in the air using HITACHI STA200. Raman spectra were carried out by Horiba LabRAM HR Evolution.

### Electrochemical Measurements

The working electrodes were prepared by mixing active materials BG@PBCFs (70 wt.%), Super P (20 wt.%), sodium carboxymethylcellulose (10 wt.%) and solvent (deionized water). The copper foil was coated evenly with the paste and then dried under vacuum at 70 °C for 12 h. Additionally, the average mass loading of the active materials was 0.5–0.8 mg cm^−2^. Electrochemical testing of BG@PBCFs as anode was carried out in CR2032 coin cell prepared in an argon‐filled glove box, using K metal and glass microfiber filter (Whatman, Grade GF/D) as counter electrode and separator, respectively. The electrolytes include 1 m KPF_6_ in a solvent of DME. The cells were charged/discharged by the CT‐4008‐5V20mA‐164 battery testing system (Neware, China) within the voltage window of 0.01–1.5 V vs K/K^+^. Cyclic voltammetry (CV) tests were performed on the CHI660e (Ch instruments, China) electrochemical workstation between 0.01 and 1.5 V at a scanning rate of 0.1 mV s^−1^. EIS spectra were achieved on the CHI660E electrochemical workstation within a frequency range from 0.01 Hz to 100 kHz.

The PTCDA|| BG@PBCFs full PIBs (with a cut‐off voltage window of 1–3.5 V) were prepared with pre‐activated BG@PBCFs anode and commercial PTCDA cathode. The PTCDA cathode was fabricated by mixing PTCDA (70 wt.%), Super P (20 wt.%), and sodium carboxymethylcellulose (10 wt.%) on an aluminum foil. The electrolyte was the same as that used in half cells. The separator was Whatman glass fiber (GF/D).

### Computational Details

The density functional theory (DFT) calculations were performed using the Vienna Ab initio Simulation Package (VASP), with the generalized gradient approximation (GGA) Perdew–Burke–Ernzerhof (PBE) functional to describe electron exchange and correlation. The projector‐augmented plane wave (PAW) potentials were used to describe the core‐valence electron interaction and take valence electrons into account using a plane wave basis set with a kinetic energy cutoff of 450 eV. Partial occupancies of the Kohn−Sham orbitals were allowed using the Gaussian smearing method and a width of 0.05 eV. The electronic energy was considered self‐consistent when the energy change was smaller than 10^−5^ eV. A geometry optimization was considered convergent when the force change was smaller than 0.02 eV Å^−1^.

The slab structure of G is composed of a mono‐layer graphite. The slab structure of NC is composed of a mono‐layer graphite doped with N of a supercell with a 15 Å vacuum separation, and the slab structures of Bi‐NC is composed of a mono‐layer graphite doped with N‐Bi of a supercell with a 15 Å vacuum separation. Based on the detection of Bi (012), Bi (101), and Bi (200) crystal planes in the XRD patterns, and the lattice structures observed in HRTEM images, an N‐Bi simulation model was constructed. A k‐points sampling of 2 × 3 × 1 with Monkhorst‐Pack scheme was used in all calculations and all calculations were considered the spin polarization effect.

For the single K and N adsorption on substrate, take K as an example, the adsorption energy of E_ads_ was defined as E_ads_ = E_tot_ ‐E_sub_ – E_K_, where E_tot_ and E_sub_ are the total energies of substrate after and before a single K adsorption, and E_K_ is the energy of each K atom in the bulk K metal. The diffusion of K atom on the substrate was studied by using the CI‐NEB method.

## Conflict of Interest

The authors declare no conflict of interest.

## Author Contributions

X.M. and W.W. conceived and supervised the research. B.Y., X.Z., and Y.C. designed and conducted the experiments. S.K., S.Y., R.M., Y.L., and Z.H. performed most of the experiments, data analysis, and discussions. B.Y. prepared the original draft of the manuscript. R.K., X.K., and M.F. reviewed and edited the manuscript. All authors discussed the results and commented on the manuscript.

## Supporting information



Supporting Information

## Data Availability

The data that support the findings of this study are available from the corresponding author upon reasonable request.
