# Supplementary material for: Bi‐Induced Few‐Layered Graphite Frameworks as Efficient Interfacial Transitions Toward Ultrafast Potassium Storage
Source: Adv Sci (Weinh). 2025 Apr 15;12(22):2416742. doi: 10.1002/advs.202416742 (PMC12165019; doi:10.1002/advs.202416742)
Supplement: Supplementary file 1 — Supporting Information [file ADVS-12-2416742-s001.docx]

Supporting Information

**Bi-induced few-layered graphite frameworks as efficient** **interfacial transitions toward ultrafast potassium storage**

*Bozhi Yang, Xin Min *, Xinyu Zhu, Shaorou Ke, Shujie Yang, Ya Chen,* *Wei Wang *, Ruiyu Mi, Yangai Liu, Zhaohui Huang, Xi Kai, Minghao Fang *, R. Vasant Kumar*

**List of Figures title**

Figure S1 Digital photographs (a) of the BG@PBCFs precursor and SEM images (b, c)

Figure S2 Digital photographs (a) of the BG@PBCFs precursor after 280°C peroxidation and SEM images (b, c)

Figure S3 Digital photographs of the BG@PBCFs

Figure S4 Lattice spacings in Bi (202), measured after manipulation with Digital Micrograph software

Figure S5 Lattice spacings in Bi (012), measured after manipulation with Digital Micrograph software

Figure S6 Lattice spacings in Bi (101), measured after manipulation with Digital Micrograph software

Figure S7 Lattice spacings in graphite (101), measured after manipulation with Digital Micrograph software (left)

Figure S8 Lattice spacings in graphite (002), measured after manipulation with Digital Micrograph software (down)

Figure S9 Lattice spacings in graphite (002), measured after manipulation with Digital Micrograph software

Figure S10 SEM images of the BCFs

Figure S11 SEM images of the PCFs

Figure S12 TEM images of the PCFs

Figure S13 N_2_ adsorption-desorption isotherm and Pore sized distribution of BCFs

Figure S14 Content of Bi element in samples with different Bi (NO_3_)_3_·5H_2_O addition amounts

Figure S15 SEM of BG@PBCFs with different amounts of added Bi (NO_3_)_3_·5H_2_O a) 5 *wt*% and b)7.5 *wt*%

Figure S16 Rate performance of BG@PBCFs with different Bi content

Figure S17 Digital images of spinning solutions with different amounts of added Bi (NO_3_)_3_·5H_2_O (10 *wt*%, 15 *wt*%, and 20 *wt*%)

Figure S18 Digital images and SEM of fiber with different amounts of added Bi (NO_3_)_3_·5H_2_O (15 *wt*%, and 20 *wt*%)

Figure S19 Rate performance of BG@PBCFs with different PAN/PMMA ratios

Figure S20 XRD patterns of different BG@PBCFs annealing temperature and time

Figure S21 SEM images of different BG@PBCFs annealing temperature and time

Figure S22 Rate capability of different BG@PBCFs annealing temperature and time

Figure S23 a) CV curves of BG@PBCFs at various scan rates. b) The determination of the b-value

Figure S24 a) Contribution ratios of capacitive- and diffusion-controlled capacities at

different scan rates. b) Contributions of the capacitive and diffusion at a scan rate of 2 mV s^-1^

Figure S25 GITT curves

Figure S26 Log D_K+_ values of discharge

Figure S27 Log D_K+_ values of charge

Figure S28 Bi (012), (101) and (200) models and adsorption models of N on Bi (012), (101), (200) and C surfaces. (Orange ball: Bi, Blue ball: N, Brown ball: C)

Figure S29 Energy of N on Bi (012), (101), (200) and C surfaces

Figure S30 Mechanism of K-ion transport within Bi nanoparticles

Figure S31 Ex situ SEM images of BCFs after different cycles at 10A g^-1^

Figure S32 a) EIS curves of BG@PBCFs after different cycles at 10A g^-1^. b) Corresponding Rel and Rct values

Figure S33 Cycling performance of PTCDA at 1 A g^-1^

Figure S34 XRD patterns of Bi Particle

Figure S35 SEM images of Bi Particle

**List of Tables title**

Table S1. The designation of BG@PBCFs samples prepared under different annealing conditions

Table S2. Electrochemical properties comparison

Table S3. Electrochemical properties comparison of full cell


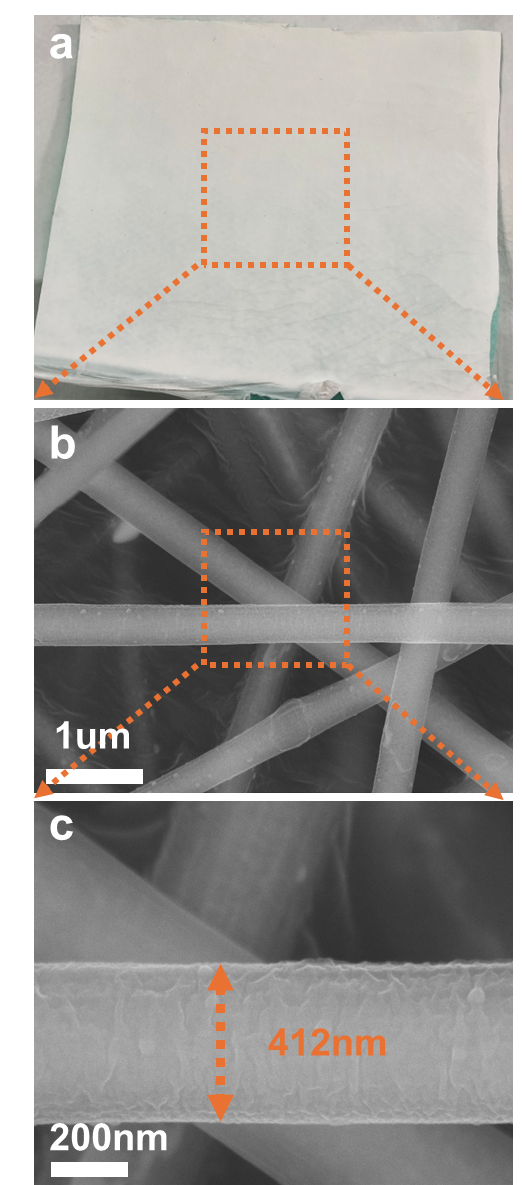


Figure S1 Digital photographs (a) of the BG@PBCFs precursor and SEM images (b, c)


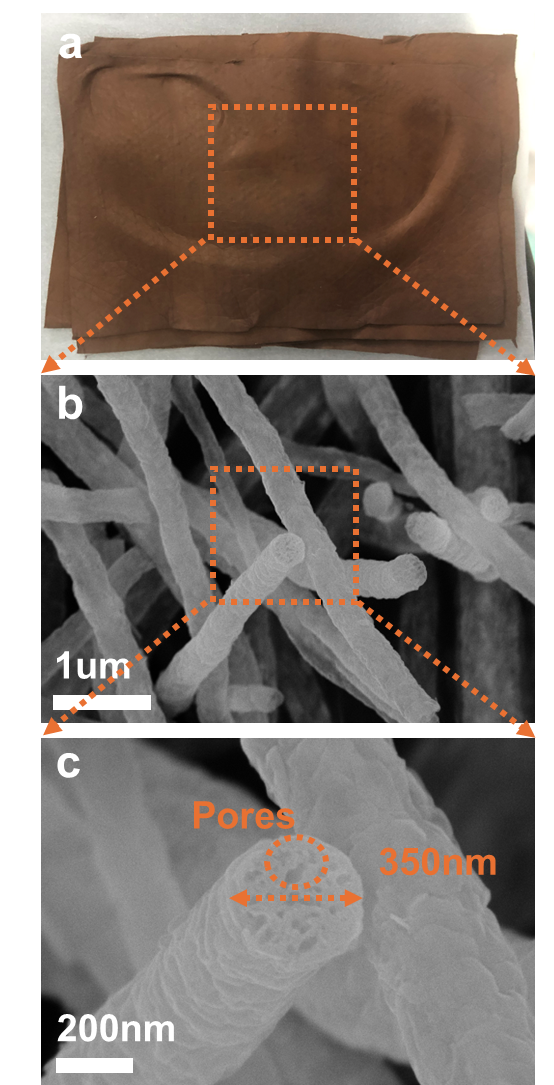


Figure S2 Digital photographs (a) of the BG@PBCFs precursor after 280°C peroxidation and SEM images (b, c)


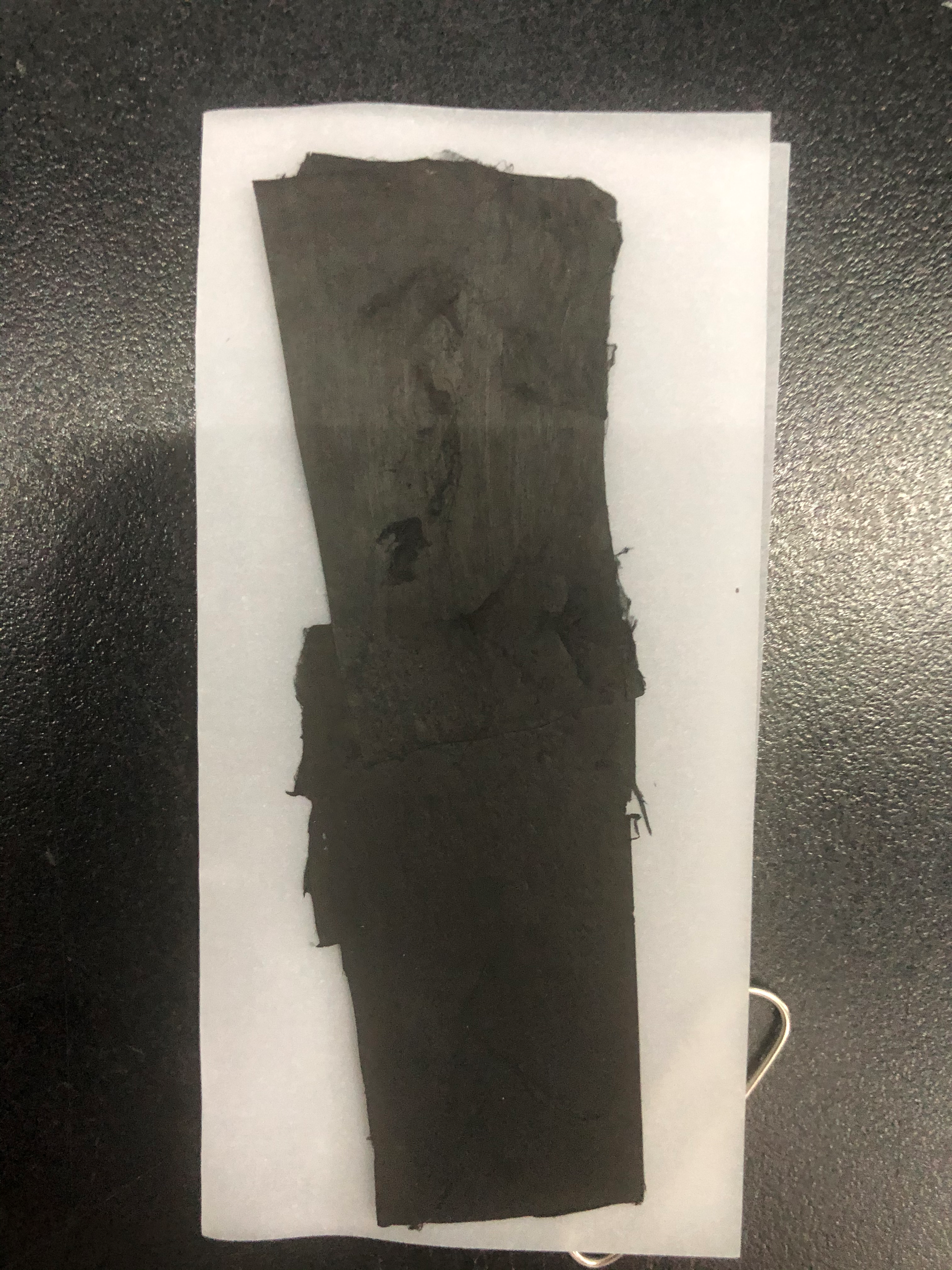


Figure S3 Digital photographs of the BG@PBCFs


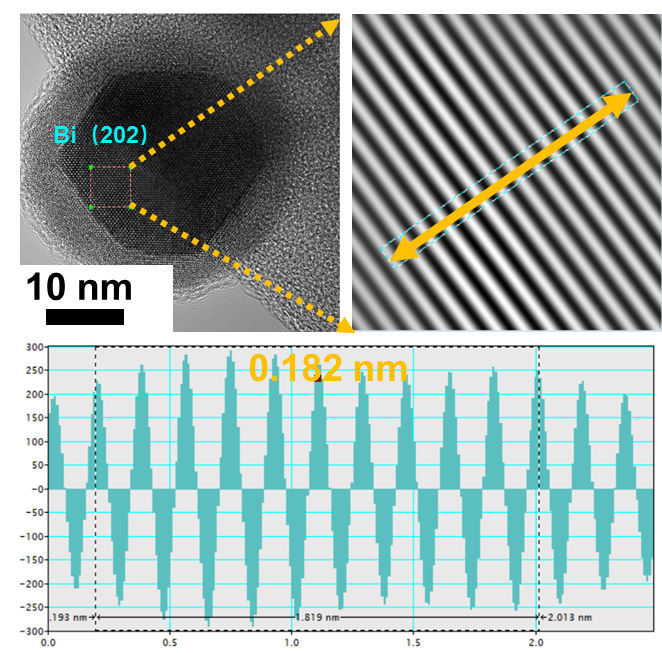


Figure S4 Lattice spacings in Bi (202), measured after manipulation with Digital Micrograph software.


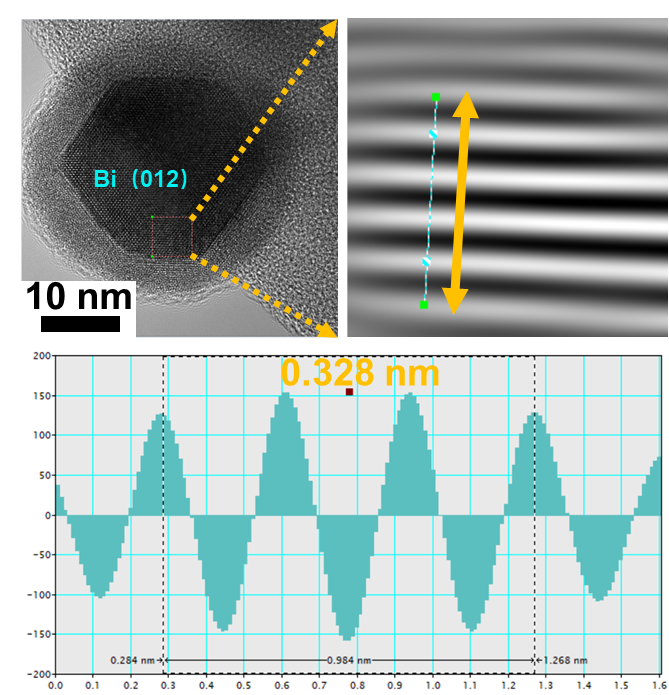


Figure S5 Lattice spacings in Bi (012), measured after manipulation with Digital Micrograph software


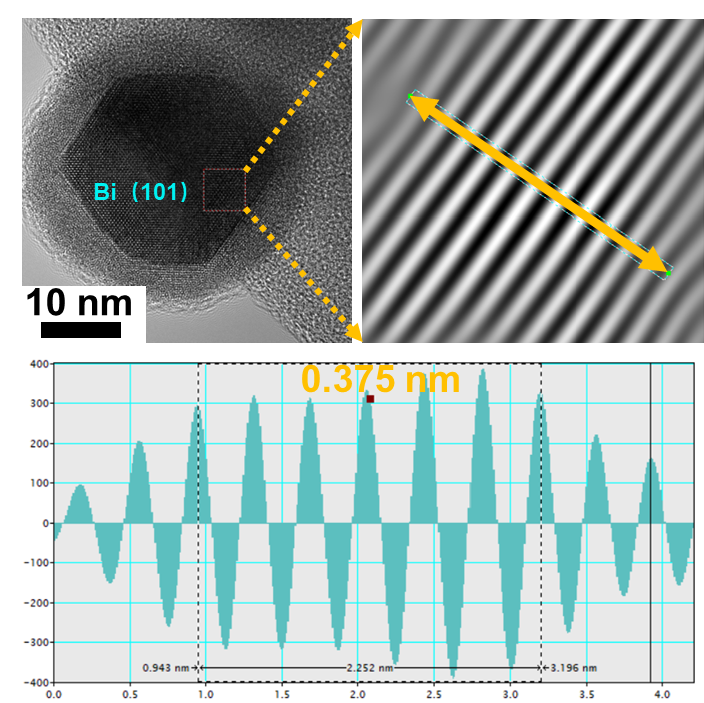


Figure S6 Lattice spacings in Bi (101), measured after manipulation with Digital Micrograph software


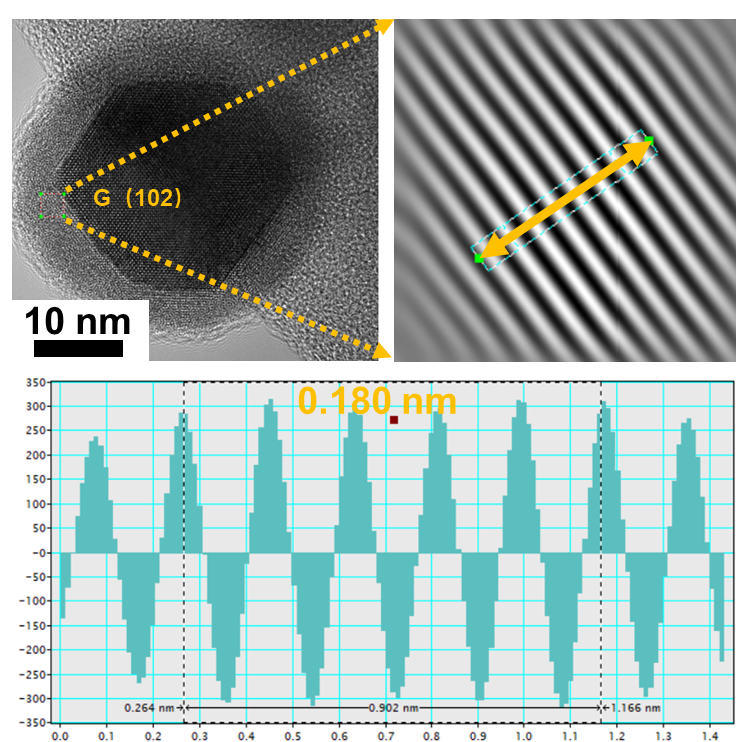


Figure S7 Lattice spacings in graphite (101), measured after manipulation with Digital Micrograph software (left)


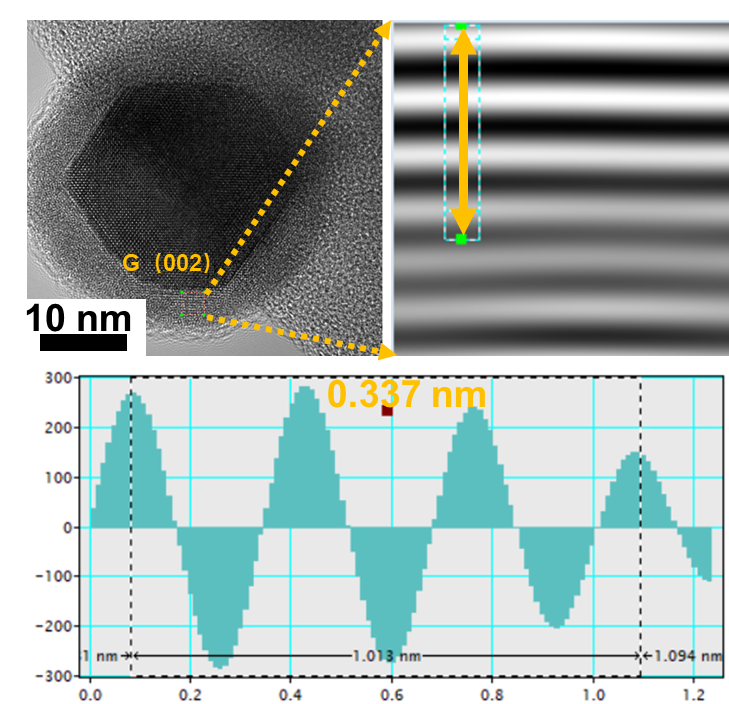


Figure S8 Lattice spacings in graphite (002), measured after manipulation with Digital Micrograph software (down)


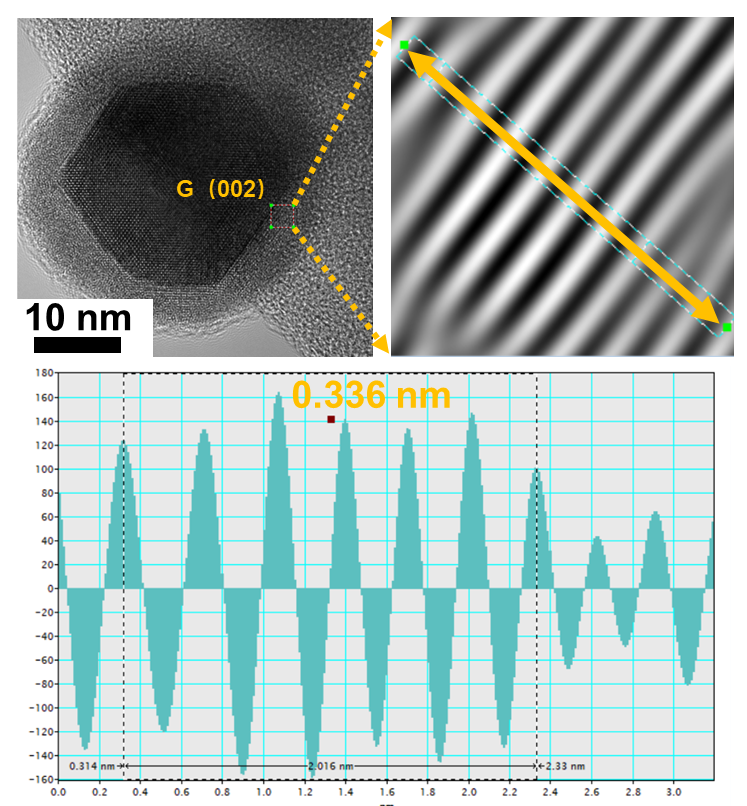


Figure S9 Lattice spacings in graphite (002), measured after manipulation with Digital Micrograph software


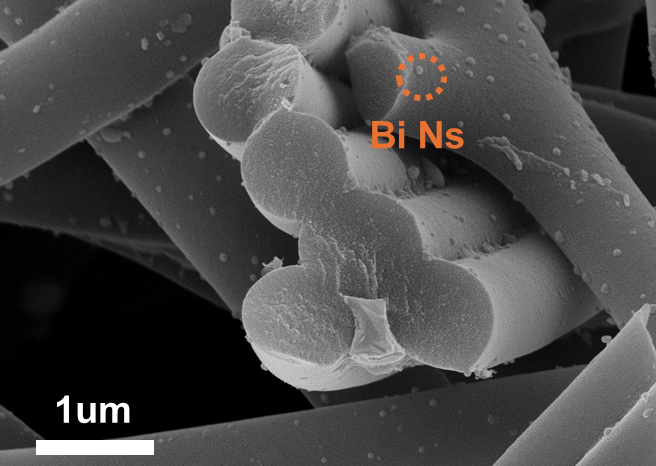


Figure S10 SEM images of the BCFs


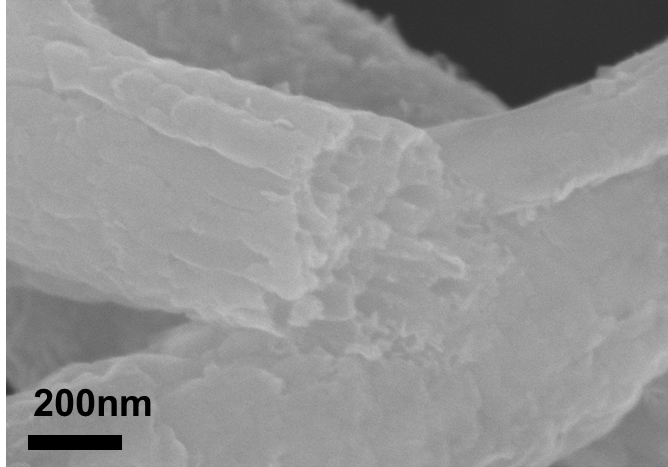


Figure S11 SEM images of the PCFs


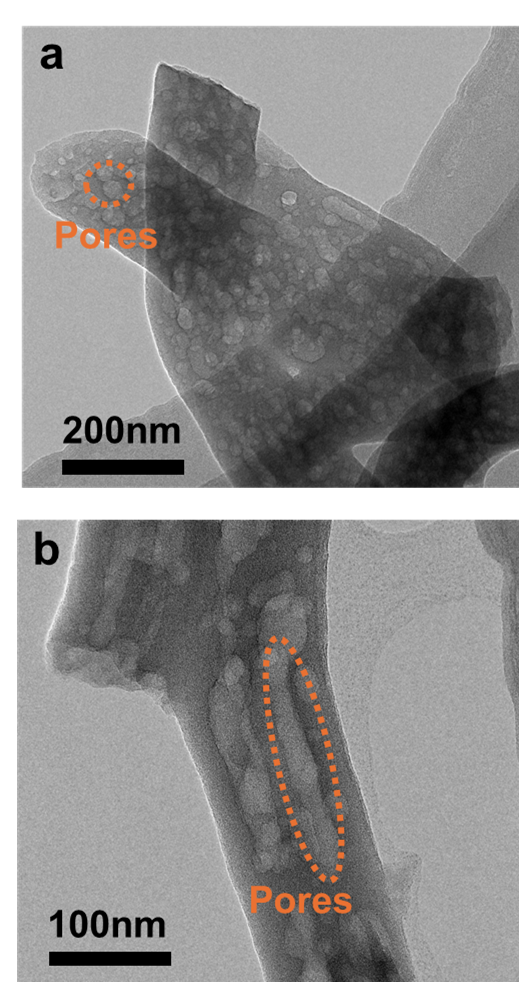


Figure S12 TEM images of the PCFs

Figure S13 N_2_ adsorption-desorption isotherm and Pore sized distribution of BCFs

Figure S14 Content of Bi element in samples with different Bi (NO_3_)_3_·5H_2_O addition amounts


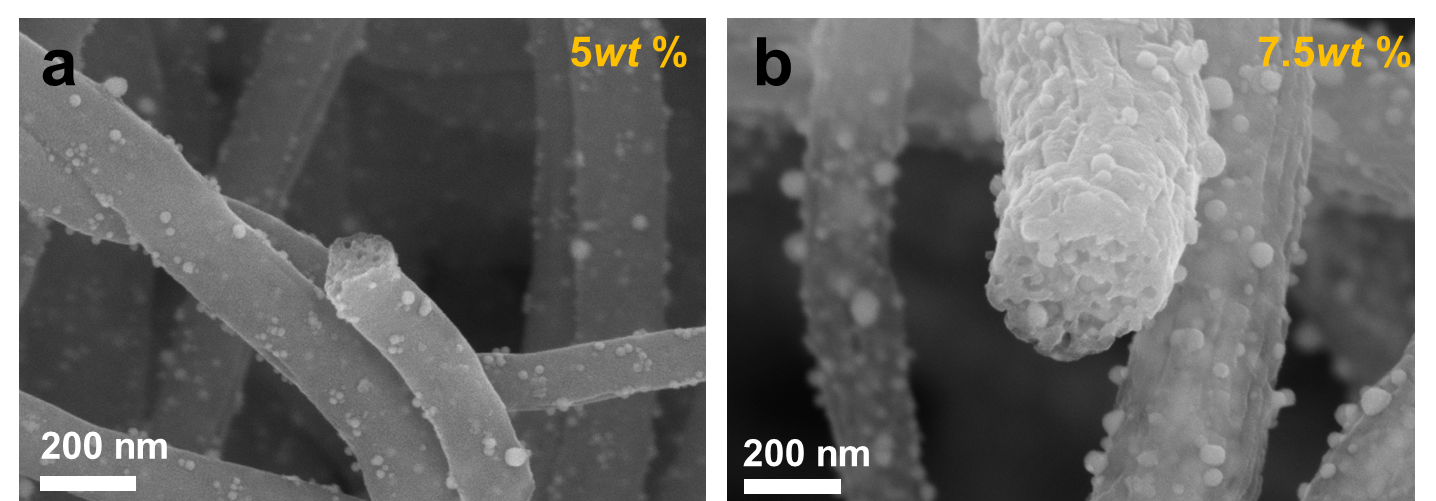


Figure S15 SEM of BG@PBCFs with different amounts of added Bi (NO_3_)_3_·5H_2_O a) 5 *wt*% and b)7.5 *wt*%

Figure S16 Rate performance of BG@PBCFs with different Bi content


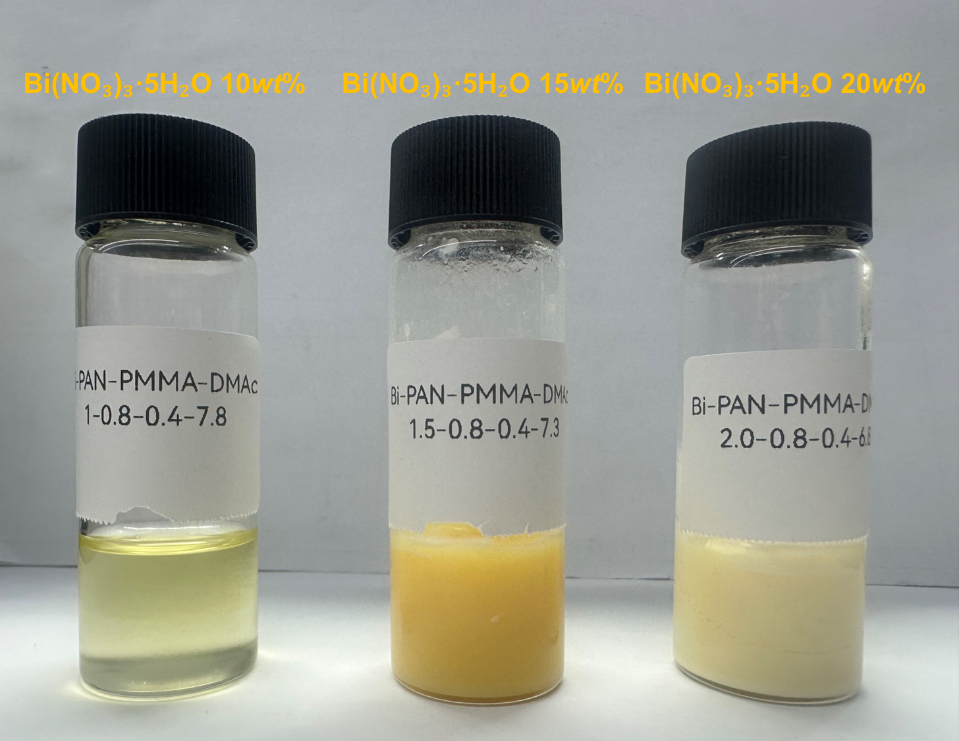


Figure S17 Digital images of spinning solutions with different amounts of added Bi (NO_3_)_3_·5H_2_O (10 *wt*%, 15 *wt*%, and 20 *wt*%)


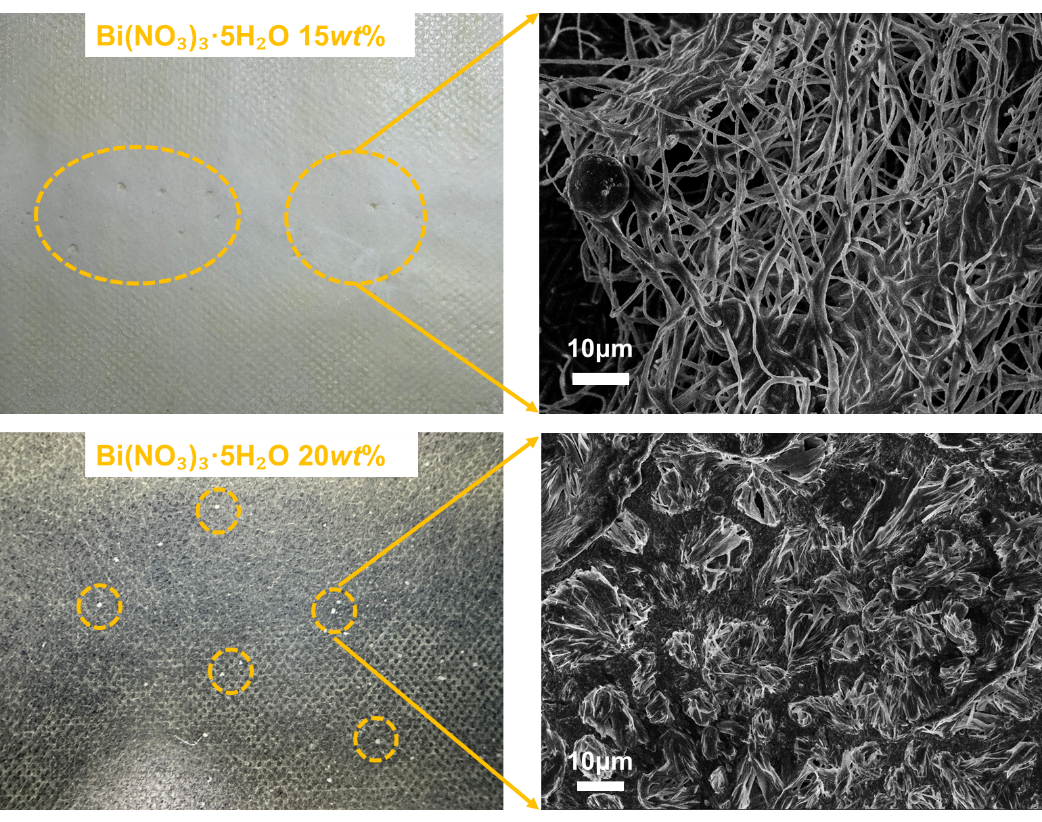


Figure S18 Digital images and SEM of fiber with different amounts of added Bi (NO_3_)_3_·5H_2_O (15 *wt*%, and 20 *wt*%)


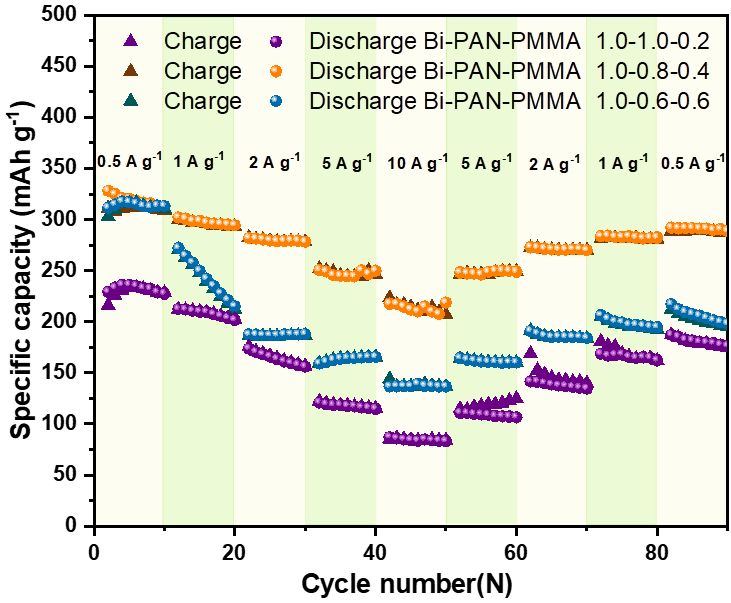


Figure S19 Rate performance of BG@PBCFs with different PAN/PMMA ratios

Figure S20 XRD patterns of different BG@PBCFs annealing temperature and time


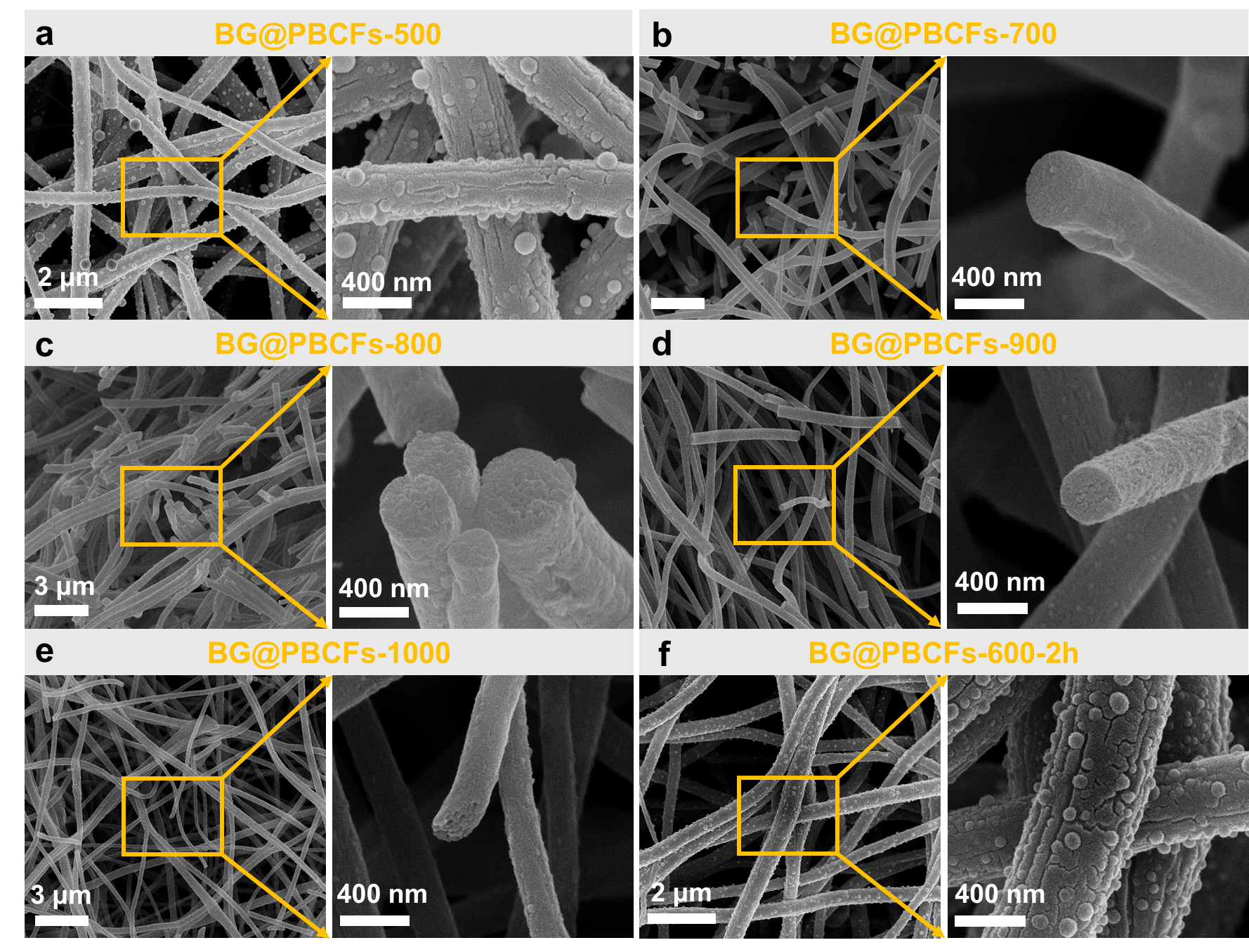


Figure S21 SEM images of different BG@PBCFs annealing temperature and time

Figure S22 Rate capability of different BG@PBCFs annealing temperature and time


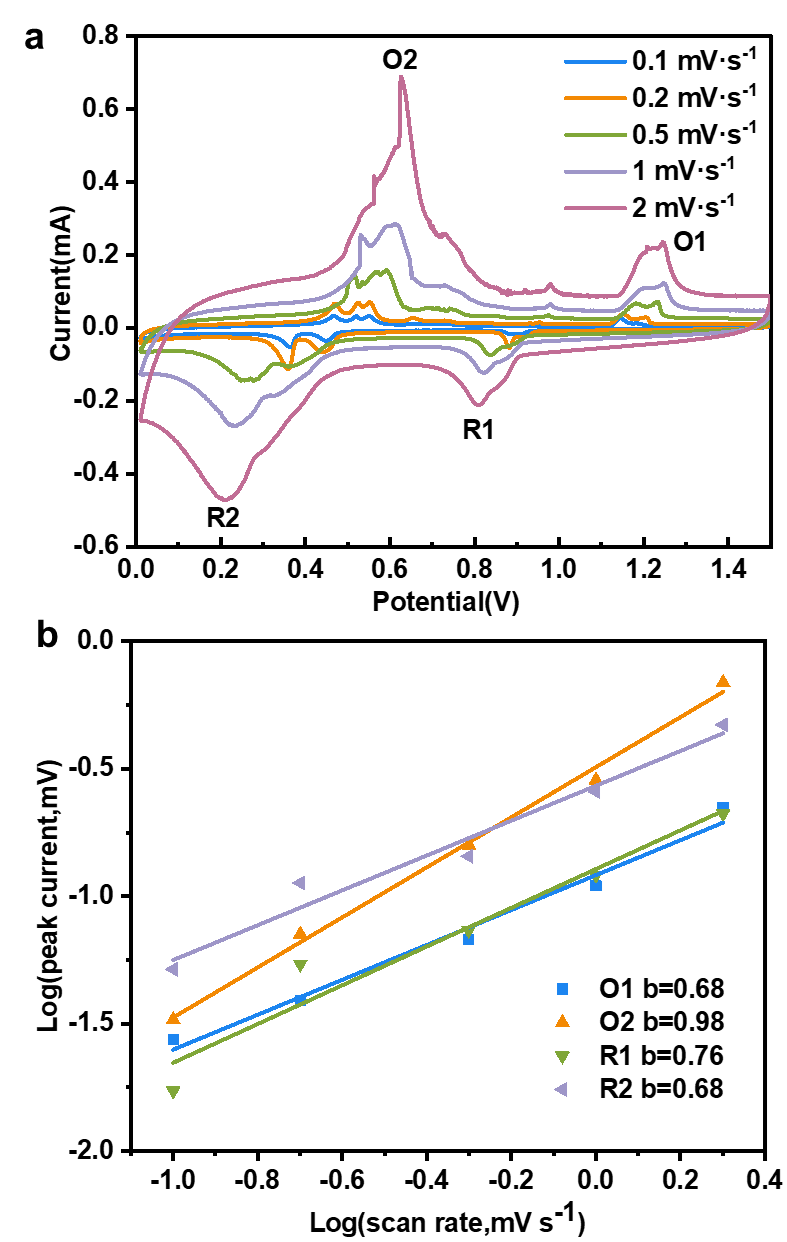


Figure S23 a) CV curves of BG@PBCFs at various scan rates. b) The determination of the b-value


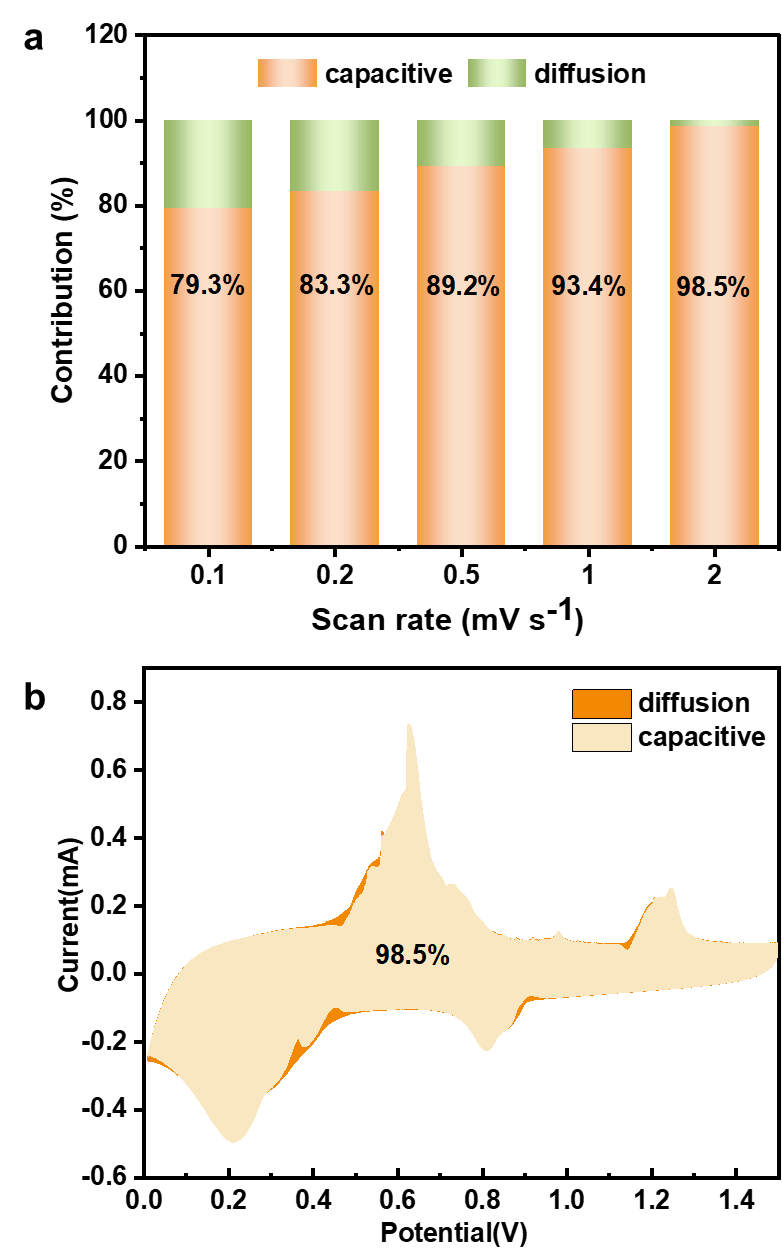


Figure S24 a) Contribution ratios of capacitive- and diffusion-controlled capacities at

different scan rates. b) Contributions of the capacitive and diffusion at a scan rate of 2 mV s^-1^

The CV curves at various scan rates (0.1−2 mV s^-1^) hold similar shapes (Figure S23 a), demonstrating fast kinetics and small polarization. Generally, the scan rate (v) and current (i) are obedient to the following relationship

$i=av^{b}$ （S1）

where a and b are fitting constants, and the b value can be acquired by the slope of log(i) vs.log(v) plots. Generally, the b values of 0.5 and 1.0 represent the completely diffusion-controlled process and capacitive-controlled behavior, respectively. [1] From Figure S23 b, the calculated b values are 0.68 (O1), 0.98(O2), 0.76 (R1) and 0.68 (R2), respectively, revealing the capacitive-controlled kinetics for BG@PBCFs Additionally, the relative contribution of each fraction from the mixed behaviors at specific potential (V) can be quantitatively evaluated by the following equation [2].

$i\left( V \right)=k_{1}v+k_{2}v^{1/2}$ （S2）

where k_1_v shows the capacitance-contribution and k_2_v^1/2^ stands for the diffusion-contribution. As an example, the capacitance-contribution was calculated to be 98.6% for BG@PBCFs at 2 mV s^−1^ as illustrated by the shaded area (Figure S24 a). As shown in Figure S24 b, the proportions of the capacitance-contribution gradually improved from 79.3% to 98.5% with the scan rates increasing from 0.2 to 1 mV s^−1^. Therefore, the charge storage in BG@PBCFs is dominated by the capacitance contribution.

Figure S25 GITT curves

Figure S26 Log D_K+_ values of discharge

Figure S27 Log D_K+_ values of charge


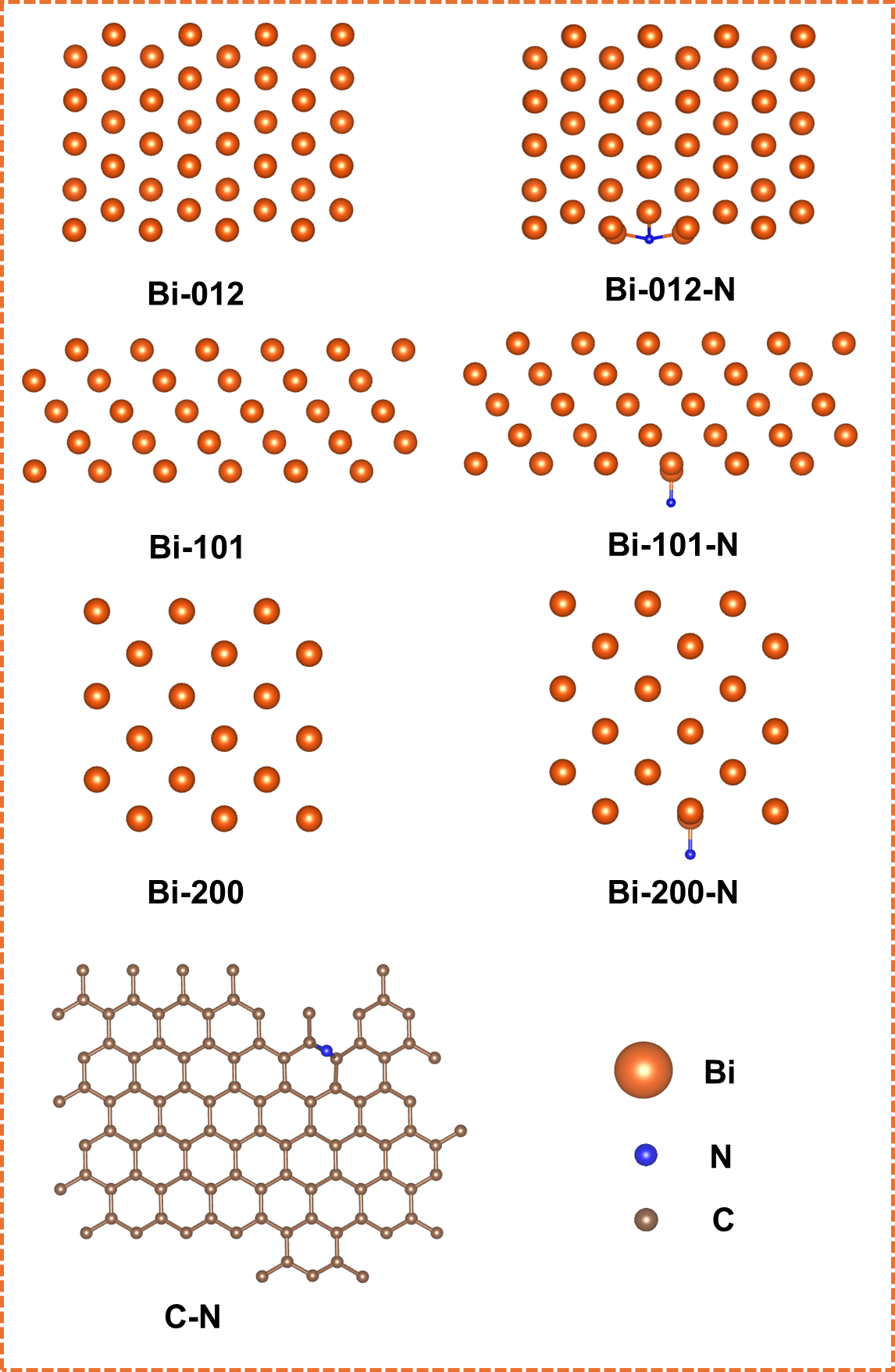
Figure S28 Bi (012), (101) and (200) models and adsorption models of N on Bi (012), (101), (200) and C surfaces. (Orange ball: Bi, Blue ball: N, Brown ball: C)

Figure S29 Energy of N on Bi (012), (101), (200) and C surfaces


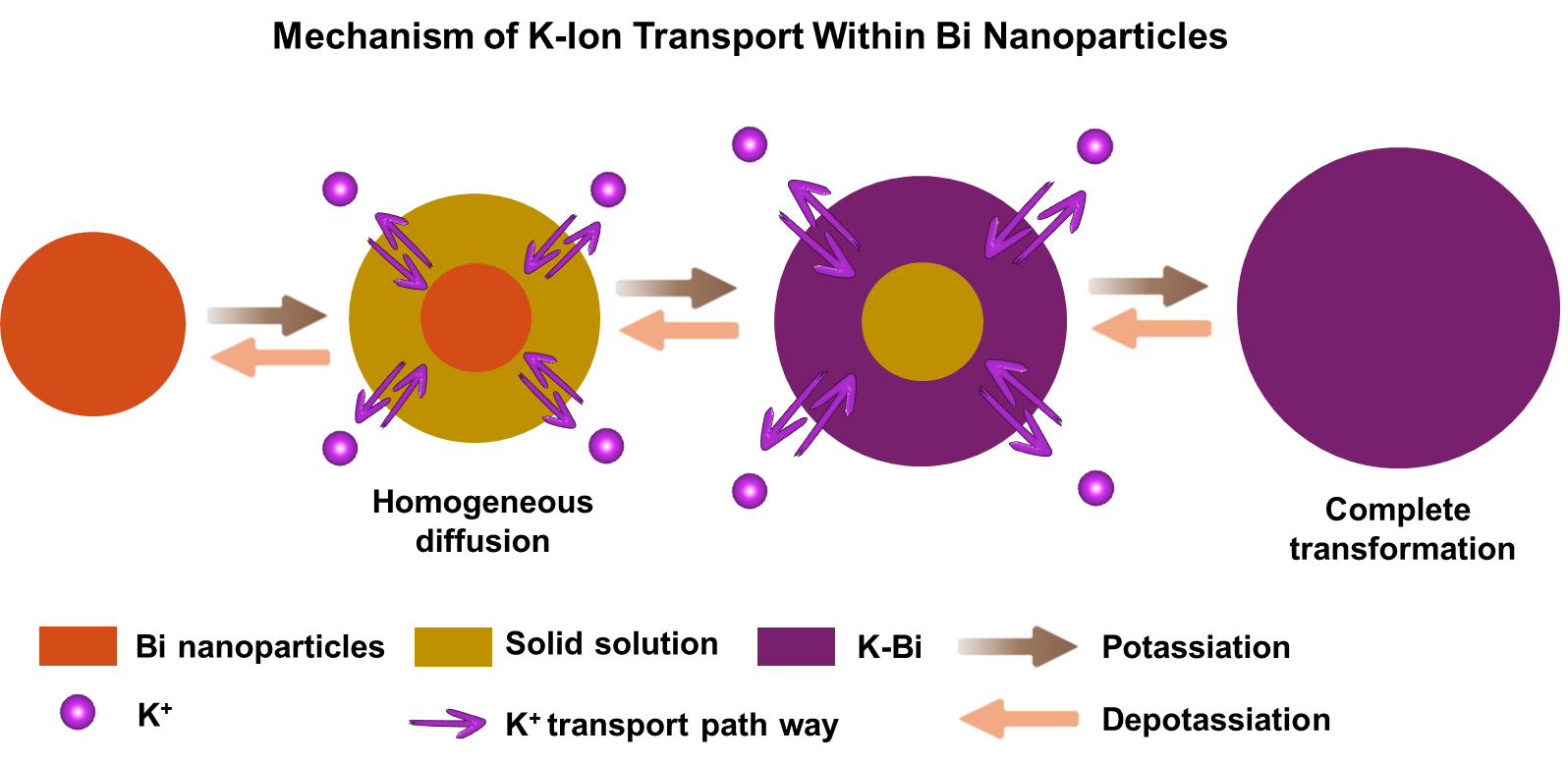


Figure S30 Mechanism of K-ion transport within Bi nanoparticles


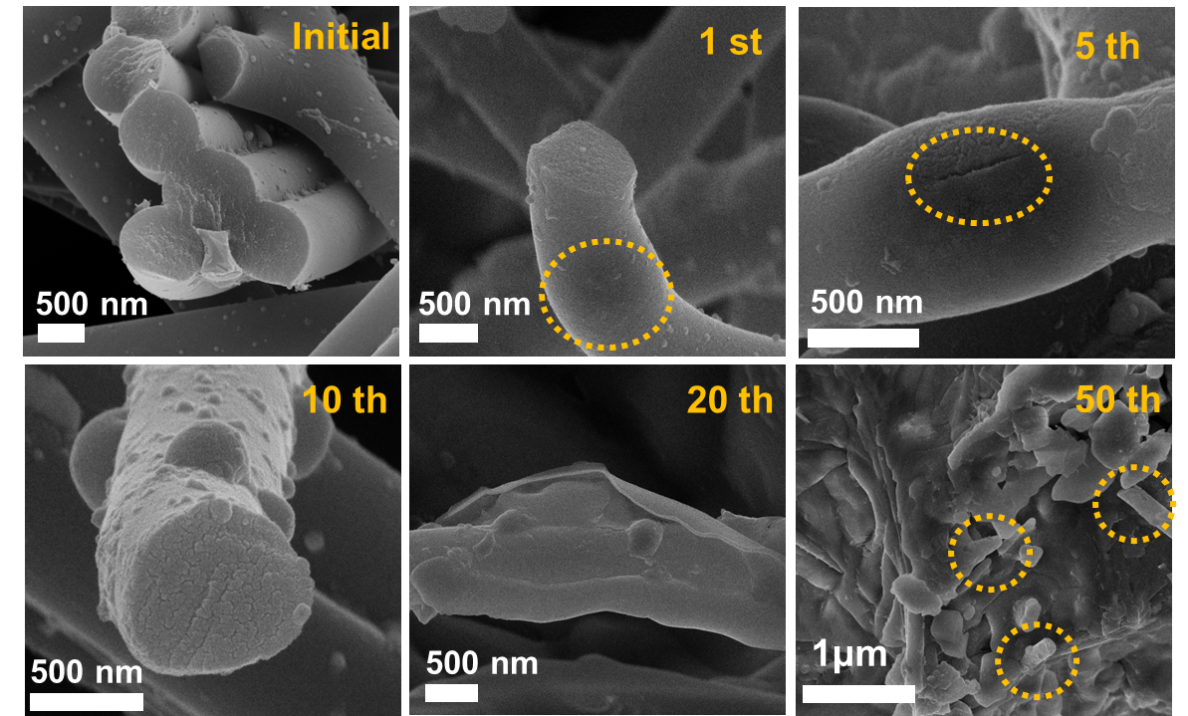


Figure S31 Ex situ SEM images of BCFs after different cycles at 10A g^-1^


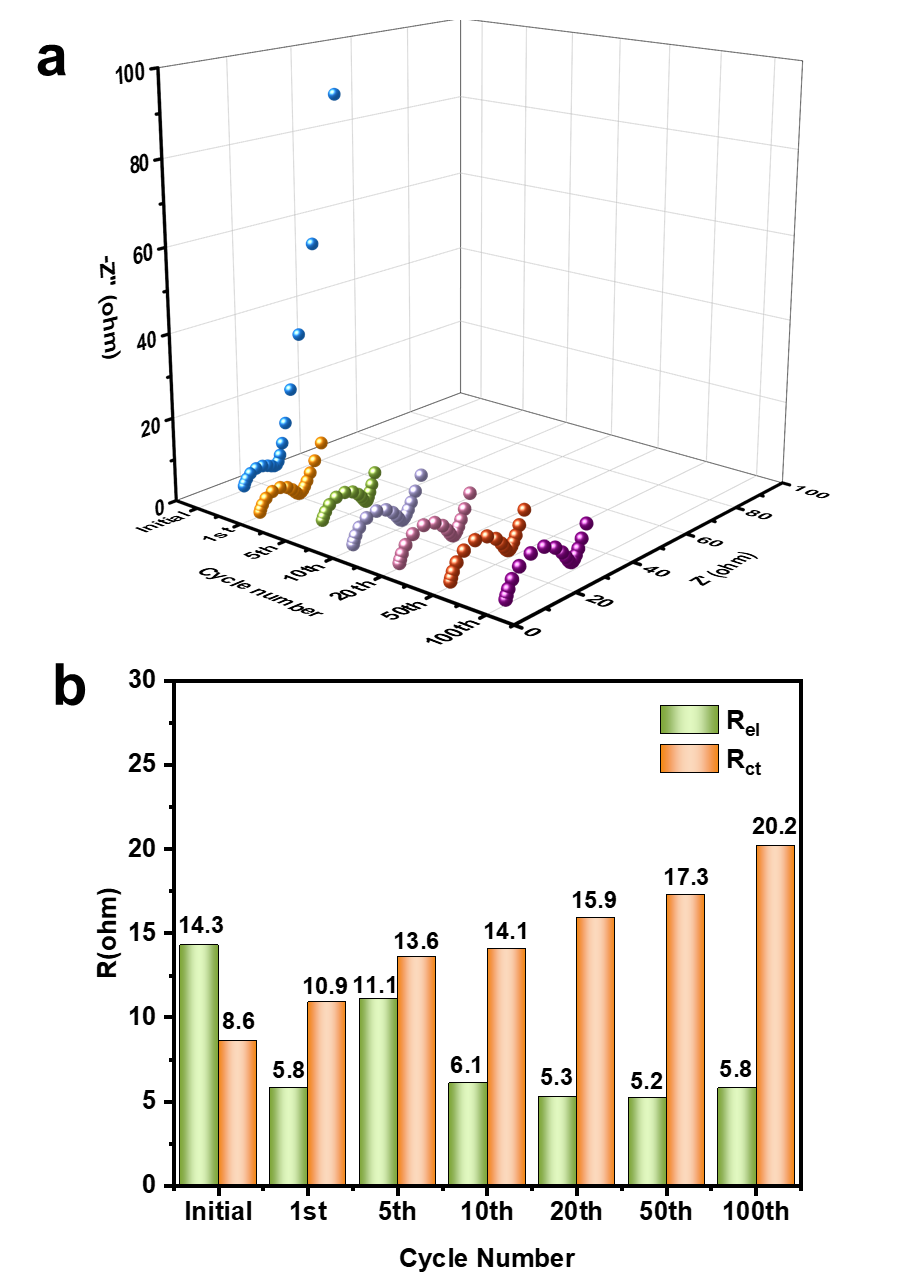


Figure S32 a) EIS curves of BG@PBCFs after different cycles at 10A g^−1^.b) Corresponding R_el_ and R_ct_ values

Figure S33 Cycling performance of PTCDA at 1 A g^-1^.

Figure S34 XRD patterns of Bi Particle


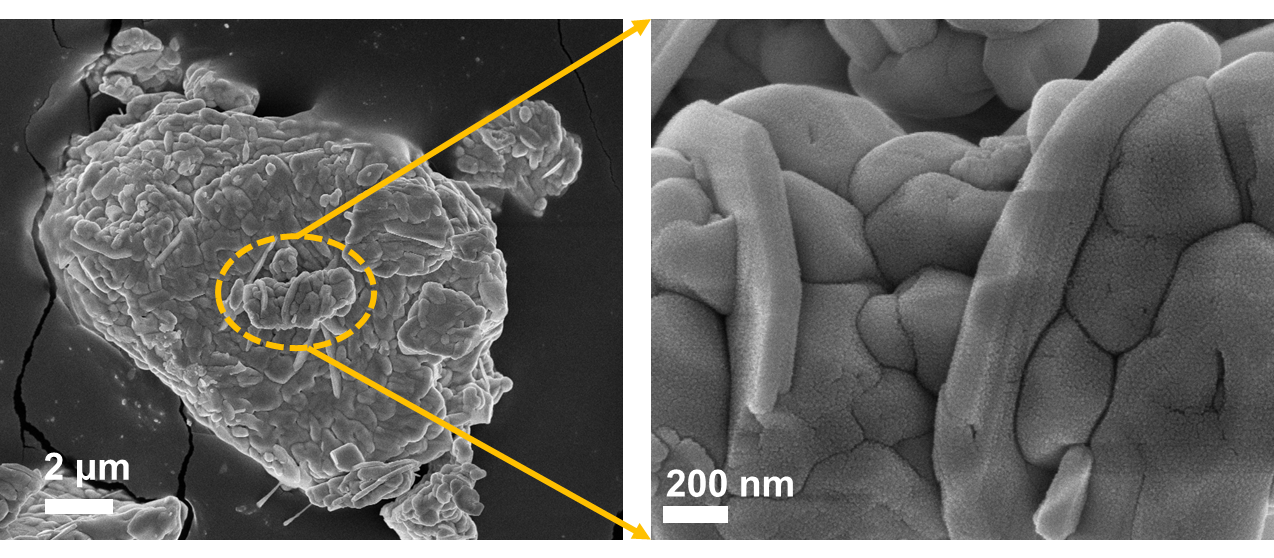


Figure S35 SEM images of Bi Particle

Table S1. The designation of BG@PBCFs samples prepared under different annealing conditions

| **Sample name** | **Temperature/℃** | **Time/h** |
| --- | --- | --- |
| BG@PBCFs-500 | 500 | 1 |
| BG@PBCFs-600 | 600 | 1 |
| BG@PBCFs-700 | 700 | 1 |
| BG@PBCFs-800 | 800 | 1 |
| BG@PBCFs-900 | 900 | 1 |
| BG@PBCFs-1000 | 1000 | 1 |
| BG@PBCFs-600-2h | 600 | 2 |

Table S2. Electrochemical properties comparison.

| Electrode | Cycle number  (N) | | Decay ratio per cycle  (%) | Current density (A g^-1^) |
| --- | --- | --- | --- | --- |
| BG@PBCFs  (This Work) | 6000 | | 0.00278 | 10 |
| Bi@NC [3] | 1400 | | 0.013 | 5.8 |
| Bi/Bi_2_O_3_ NDs@CSs [1] | 1000 | | 0.02 | 10 |
| Bi-MOF [4] | 1200 | 0.0158 | | 0.5 |
| Bi NPs/NPC [5] | 450 | 0.03977 | | 10 |
| SPB@NC [6] | 2000 | 0.01 | | 5 |
| Bi@Void@TiO_2_-CNF [7] | 3000 | 0.00487 | | 2 |
| Bi@N-CT [8] | 1000 | 0.012 | | 3.85 |
| Bi@C [9] | 1000 | 0.038 | | 0.5 |
| H-Bi-Ti-EG [10] | 800 | 0.0087 | | 0.1 |
| (Bi-Sb)_2_S_3_@N-C [11] | 1600 | 0.00937 | | 2 |
| Bi_0.48_Sb_1.52_Se_3_@C [12] | 1000 | 0.02 | | 1 |
| 2D-Sb_0.6_Bi_0.4_ [13] | 1000 | 0.037 | | 1 |

Table S3. Electrochemical properties comparison of full cell.

| **Full cell** | | **Energy density** | **Rate capacity** |
| --- | --- | --- | --- |
| PTCDA\|\|BG@ PBCFs  (this work) | | 221 Wh kg^-1^ at 527.9 W kg^-1^ and 153 Wh kg^-1^ at 10.6kW kg^-1^ | 144 mAh g^-1^ at 10 A g^-1^ |
| Bi-N_4_-O_2_@HCR-1000\|\|MKVO [14] | | 190.8 Wh kg^-1^ at 507 W kg^-1^ | 75 mAh g^-1^ at 10 A g^-1^ |
| Mg-KVO\|\|Bi-N_3_S_1_/CNSs [15] | 153.4 Wh kg^-1^ at 400 W kg^-1^ | | 157.86 mAh g^-1^ at 5 A g^-1^ |
| MKVO\|\|Bi/Bi_2_O_3_NDs@CSs [1] | 135.9 Wh kg^-1^ at 415 W kg^-1^ | | 121.7 mAh g^-1^ at 10 A g^-1^ |
| SPB@NC\|\|KVO [6] | 177.7 Wh kg^-1^ at 883.6 W kg^-1^ | | 132.5 mAh g^-1^ at 10 A g^-1^ |
| PTCDA\|\|Bi NPs/NPC [5] | 130.7 Wh kg^-1^ at 809.8 W kg^-1^ | | 166.8 mAh g^-1^ at 5 A g^-1^ |
| Bi/Bi_3_Se_4_@CNR//KVO [16] | 190.8 Wh kg^-1^ at 507 W kg^-1^ | | 134.4 mAh g^-1^ at 5 A g^-1^ |
| Bi//PB [17] | 108.1 Wh kg^-1^ at 566 W kg^-1^ | | 32 mAh g^-1^ at 0.5 A g^-1^ |

**References**

[1] X. Liu, Z. F. Sun, Y. J. Sun, H. X. Lin, Z. S. Chen, X. X. Chen, L. Niu, Q. B. Zhang, H. Y. Li, *Advanced Functional Materials* **2023**, *33* (52), https://doi.org/10.1002/adfm.202307205.

[2] R. C. Cui, H. Y. Zhou, J. C. Li, C. C. Yang, Q. Jiang, *Advanced Functional Materials* **2021**, *31* (33), https://doi.org/10.1002/adfm.202103067.

[3] X. Y. Xiang, D. Liu, X. X. Zhu, Y. Y. Wang, D. Y. Qu, Z. Z. Xie, X. Zhang, H. Zheng, *ACS Applied Materials & Interfaces* **2022**, *14* (30), 34722, https://doi.org/10.1021/acsami.2c07606.

[4] S. Y. Li, Q. S. Zhang, H. L. Deng, S. Chen, X. H. Shen, Y. Z. Yuan, Y. L. Cheng, J. Zhu, B. A. Lu, *Small Methods* **2023**, *7* (6), https://doi.org/10.1002/smtd.202201554.

[5] X. Liu, X. Z. Yu, Y. Tong, Y. J. Sun, W. J. Mai, L. Niu, H. Y. Li, *CHEMICAL ENGINEERING JOURNAL* **2022**, *446*, https://doi.org/10.1016/j.cej.2022.137329.

[6] X. Liu, Y. J. Sun, Y. Tong, H. Y. Li, *Small* **2022**, *18* (44), https://doi.org/10.1002/smll.202204045.

[7] Z. Y. Gao, L. Han, H. Gao, J. W. Chen, Z. N. Sun, C. L. Zhu, Y. F. Zhang, J. Shi, S. G. Chen, H. L. Wang, *Journal of Materials Chemistry A* **2022**, *10* (24), 12908, https://doi.org/10.1039/d2ta01833k.

[8] H. Li, C. Zhao, Y. Yin, Y. Zou, Y. Xia, Q. An, Z. Jian, W. Chen, *Nanoscale* **2020**, *12* (7), 4309, https://doi.org/10.1039/C9NR09867D.

[9] J. Yao, C. L. Zhang, G. W. Yang, M. Sha, Y. L. Dong, Q. Fu, Y. H. Wu, H. P. Zhao, M. H. Wu, Y. Lei, *ACS Applied Materials & Interfaces* **2021**, *13* (27), 31766, https://doi.org/10.1021/acsami.1c09286.

[10] Z. Q. Li, J. Q. Wen, Y. Q. Cai, F. T. Lv, X. Zeng, Q. Liu, T. Masese, C. X. Zhang, X. S. Yang, Y. W. Ma, H. J. Zhang, Z. D. Huang, *Advanced Functional Materials* **2023**, *33* (22), https://doi.org/10.1002/adfm.202300582.

[11] L. P. Yang, L. Guo, D. Yan, Y. Wang, T. Shen, D. S. Li, M. E. Pam, Y. M. Shi, H. Y. Yang, *ACS Nano* **2023**, *17* (7), 6754, https://doi.org/10.1021/acsnano.2c12703.

[12] T. Yuan, J. T. Yan, Q. F. Zhang, Y. Su, S. H. Xie, B. A. Lu, J. Y. Huang, X. P. Ouyang, *ACS Nano* **2023**, *17* (11), 10462, https://doi.org/10.1021/acsnano.3c01260.

[13] X. Liu, X. Y. Wang, Y. R. Zhou, B. C. Wang, L. G. Zhao, H. Zheng, J. B. Wang, J. H. Liu, J. Liu, Y. Y. Li, *Advanced Materials* **2024**, *36* (11), https://doi.org/10.1002/adma.202308447.

[14] Z. Chen, H. Lin, Y. Tan, L. Niu, H. Li, *Advanced Functional Materials* **2024**, *34* (45), 2407653, https://doi.org/https://doi.org/10.1002/adfm.202407653.

[15] Y. Tan, H. Lin, Z. Chen, L. Niu, H. Li, *Journal of Energy Chemistry* **2024**, *99*, 365, https://doi.org/https://doi.org/10.1016/j.jechem.2024.07.054.

[16] Z. Chen, Y. Wu, X. Liu, Y. Zhang, L. Yang, H. Li, *Journal of Energy Chemistry* **2023**, *81*, 462, https://doi.org/https://doi.org/10.1016/j.jechem.2023.02.050.

[17] K. Lei, C. Wang, L. Liu, Y. Luo, C. Mu, F. Li, J. Chen, *Angewandte Chemie International Edition* **2018**, *57* (17), 4687, https://doi.org/https://doi.org/10.1002/anie.201801389.
